# Fully Automated Radiosynthesis of No‐Carrier‐Added [^11^C]Butanol Using the GE FASTLab 2 Module

**DOI:** 10.1002/jlcr.4158

**Published:** 2025-07-31

**Authors:** Ivan E. Wang, Jason A. Witek, Ryan J. Pakula, Bradford D. Henderson, Marianna Dakanali, Xia Shao, Peter J. H. Scott

**Affiliations:** ^1^ Department of Medicinal Chemistry, College of Pharmacy University of Michigan Ann Arbor Michigan USA; ^2^ Department of Radiology University of Michigan Ann Arbor Michigan USA; ^3^ Department of Pharmacology University of Michigan Ann Arbor Michigan USA

## Abstract

Radiolabeled alcohols have been investigated for their use in the measurement of cerebral blood flow for years. In particular, [^11^C]butanol has been employed as a freely diffusible tracer with appreciable tissue retention and good solubility in both lipid and aqueous compartments. It is an appropriate radiotracer for the assessment of blood supply and for the comparative evaluation of substrate utilization with blood flow. Herein, we describe a no‐carrier‐added [^11^C]butanol radiosynthesis using the GE FASTLab 2 to leverage the benefits of a cassette‐based workflow, and we compare it to our legacy radiosynthesis using GE TracerLab FX modules. Using the FASTLab 2, [^11^C]butanol was synthesized in 21 min with a radiochemical yield of 4%–8% (*n* = 3) and a radiochemical purity of > 90%. Synthesis on the FASTLab was fast, reliable, and comparable to syntheses using TracerLab FX modules.

## Graphical abstract

Using the FASTLab 2, [^11^C]butanol was synthesized in 21 min with a radiochemical yield of 4%–8% (*n* = 3) and a radiochemical purity of > 90%. Synthesis on the FASTLab was fast, reliable, and comparable to legacy syntheses using TracerLab FX modules.

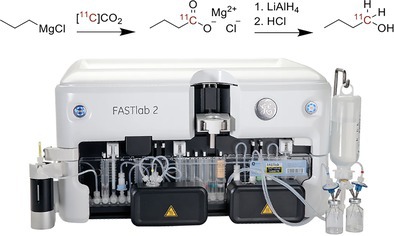

## 1 Introduction

Cerebral blood flow (CBF), blood‐brain perfusion, and blood‐brain barrier status are important parameters when studying brain function, the efficacy of drug delivery, and tumor pathophysiology at the cellular and molecular level [1], and there has been considerable interest over the years in quantifying these using positron emission tomography (PET). In particular, dysregulated fluid exchange can be measured using [^15^O]water and [^11^C]butanol. [^15^O]Water is a diffusible tracer that relies on facilitated diffusion via aquaporin (AQP4) channels to enter the brain (E_*w*_ 0.9 mL/g). Unlike [^15^O]water, which can saturate the AQP4 channels, [^11^C]butanol is a freely diffusible tracer that is not dependent on the concentration gradient (E_*diff*_ 0.96 mL/g) [2, 3, 4, 5]. Moreover, [^11^C]butanol is a perfusion tracer with a superior extraction fraction compared with [^15^O]water. When the blood‐brain barrier (BBB) is intact, the extraction fraction of water will be lower than that of [^11^C]butanol. In patients with a disrupted BBB, the extraction fraction of water will increase due to increased water extravasation into the brain [3, 4]. In CBF measurements, [^11^C]butanol has been employed with appreciable tissue retention and equally good solubility in lipid and aqueous components and therefore has gained attraction for use as a CBF agent in research and clinical studies [6]. More recently, there has been renewed interest in using [^11^C]butanol in conjunction with new total‐body PET (TBP) scanners to measure total‐body perfusion and evaluate local perturbations in flow because of physiologic stressors and disease [7, 8].

**SCHEME 1:**
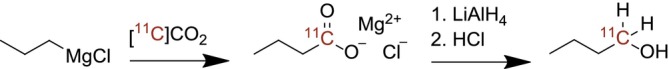
Radiosynthesis of [^11^C]butanol.

Radiosynthesis of no‐carrier‐added [^11^C]butanol has previously been established using the carbonylation of an organoborane; however, this process is long (60 min), and the radiochemical yields (RCY) typically low (33%–71% non‐decay corrected yield) [9]. Modern syntheses use the carboxylation of *n*‐propyl magnesium chloride (*n*‐PrMgCl) with [^11^C]CO_2_, followed by the reduction of the resulting carboxylate salt with LiAlH_4_ (Scheme 1) [9, 10]. Reported procedures were often complicated and limited to manual or semi‐automated approaches. Therefore, an automated method, using a commercially available synthesis module, is in high demand [9, 10, 11]. Herein, we have demonstrated a new procedure using a cassette‐based synthesis on the GE FASTLab 2 synthesizer (hereafter referred to as FASTLab). This approach reduces cleaning requirements and day‐of‐synthesis setup time and eliminates the possibility of cross‐contamination and the need for a non‐standard module configuration to make [^11^C]butanol. To determine the robustness of the new method, we have compared our FASTLab synthesis to our established procedures using GE TracerLab FX modules (FX_C‐Pro_, FX_M_, and FX_FN_) [12]. The simplified process on the FASTLab, using pre‐sealed vials of the air sensitive Grignard and lithium aluminum hydride (LAH) reagents, improves reproducibility and compliance for routine radiopharmaceutical production under current good manufacturing practice (cGMP). Additionally, a reliable and faster quality control HPLC method has been developed to further improve the production of [^11^C]butanol at our facility for use in ongoing clinical studies [13] under the Radioactive Drug Research Committee approval (limit of injection = 25 mg per study, 50 mg [or 5000 ppm] per day).

## 2 Materials and Methods

### 2.1 General Considerations

FASTLab 2, TracerLab FX_M_, and TracerLab FX_C‐Pro_ synthesizer modules (GE Healthcare, Uppsala, Sweden) were used for the radiosynthesis. The chemical reagents and solvents, 2 M *n*‐propyl magnesium chloride solution in diethyl ether, 1 M lithium aluminum hydride (LAH) in diethyl ether, and diethyl ether (≥ 99.7% purity) were purchased from Sigma‐Aldrich, St. Louis, MO, USA. [^11^C]CO_2_ was produced by proton bombardment of a 0.5% O_2_ in N_2_ target via the ^14^N(p,α)^11^C nuclear reaction using a GE Healthcare PETtrace 800 cyclotron. The unlabeled reference standard [^12^C]butanol was also purchased from Sigma‐Aldrich. The solid phase extraction (SPE), C18 plus short (360 mg), C18 plus long (820 mg), C18 6 cc Vac (1 g), and C18 12 cc Vac (2 g) cartridges were purchased from Waters, Milford, MA, USA. GE FASTLab developer kits and components were purchased from GE Healthcare. The radiolabeled [^11^C]butanol was characterized by a Shimadzu LC2010A HPLC (Kyoto, Japan) with a UV detector and a Bioscan/Eckert and Ziegler radioactivity detector (Eckert & Ziegler Radiopharma Inc., Hopkinton, MA, USA). Residual solvent analysis was performed using undiluted samples on a Shimadzu GC‐2010 (Kyoto, Japan). HPLC analysis was conducted using a Phenomenex Luna C18(2), 5 μm, 100 Å, 250 × 4.6 mm column. Samples (10 μL) obtained from the FASTLab were diluted with 90 μL of 20% acetonitrile in the autosampler prior to injection.

### 2.2 Radiosynthesis of [^11^C]Butanol on FASTLab

**FIGURE 1:**
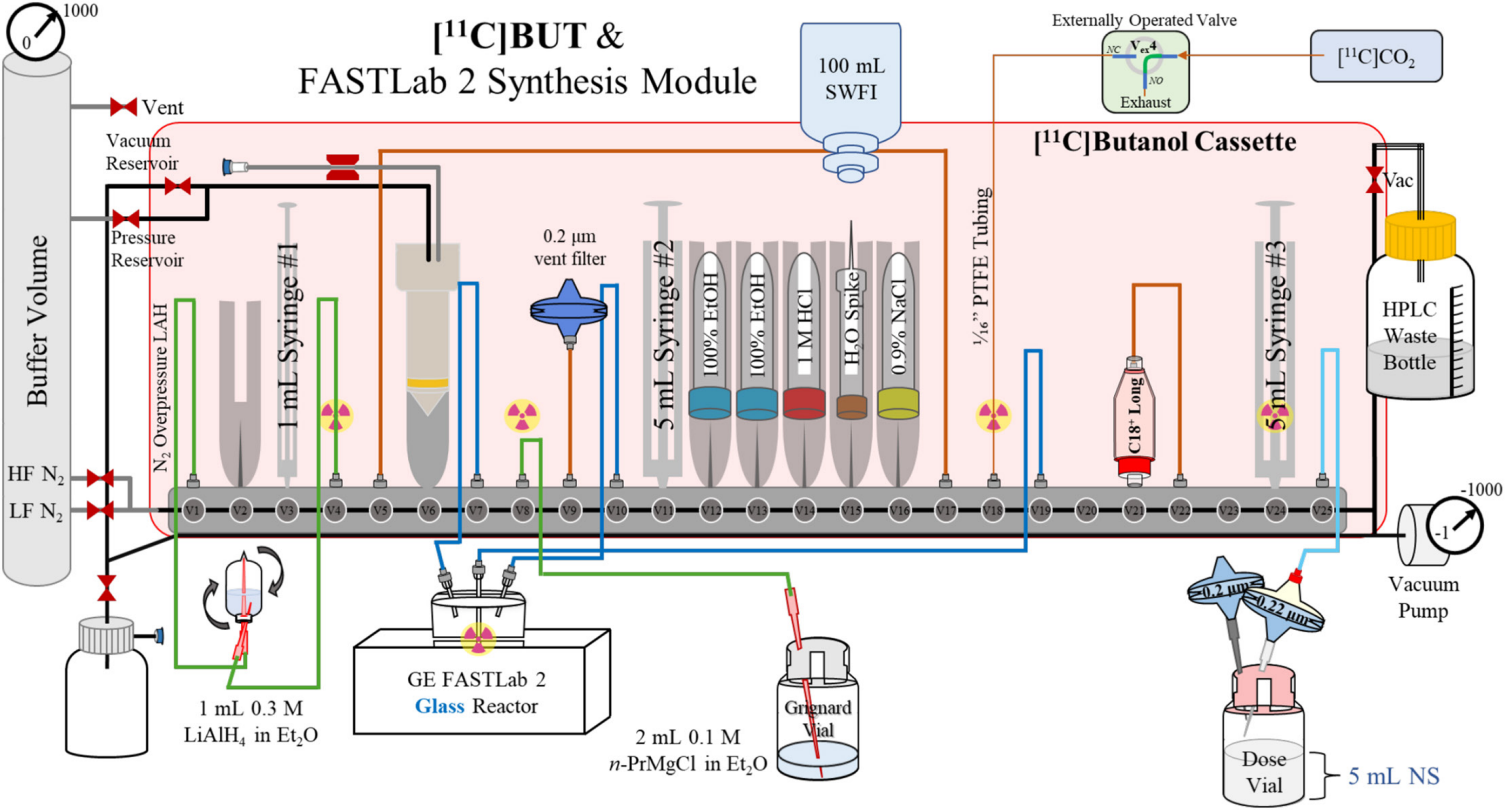
FASTLab cassette setup for the synthesis of [^11^C]butanol. Highlighted in green are lines used to connect to the inverted LAH vial, and the line used to add the Grignard when prompted by the program. In dark blue are the lines corresponding to the GE FASTLab glass reactor. NS—0.9% sodium chloride, USP.

[^11^C]Butanol was synthesized on a FASTLab synthesis module using the developer cassette configuration illustrated in Figure 1 and listed in Table 1 and according to the timelist provided as Supporting Information (Figure S1). Briefly, the FASTLab cassette was configured such that the LAH vial and Grignard can be added after the start of the program to limit exposure to moisture. The Grignard solution was diluted under inert (nitrogen) atmosphere by using 100 μL of 2 M *n*‐PrMgCl with 1900 μL ether to make a 0.1 M solution and added to a sealed vial that was preflushed with nitrogen gas. The LAH solution was also diluted under inert atmosphere by using 300 μL of 1 M LAH with 700 μL ether to make a 0.3 M solution and added to a separate sealed vial that was also preflushed with nitrogen gas.

**TABLE 1:** FASTLab cassette configuration and components.

Position/vial	Component
V1	42‐cm‐long silicone tubing connected to an 18 G 1.5″ needle inserted into an inverted 2 mL vial (LAH vial) reaching **above** the solvent line (only added once prompted by the timelist, to minimize potential reaction with air)
V2	*Empty, no vial*
V3	1‐mL syringe (syringe #1)
V4	42‐cm‐long silicone tubing connected to an 18 G 1.5″ needle inserted into an inverted 2‐mL vial (LAH vial) reaching the **inner septa** of the inverted vial (only added once prompted by the timelist, to minimize potential reaction with air)
V5	42‐cm‐long silicone tubing connected to V17
V6	*Conical reservoir (not used)*
V7	42‐cm‐long silicone tubing connected to glass reactor
V8	42‐cm‐long silicone tubing connected to an 18 G 3.5″ spinal needle inserted into a 10‐mL vial (Grignard vial) reaching the **bottom** of the vial (only added once prompted by the timelist, to minimize potential reaction with air and evaporation)
V9	14‐cm‐long silicone tubing connected to a 0.2‐μm filter
V10	14‐cm‐long silicone tubing connected to glass reactor
V11	5‐mL syringe (syringe #2)
V12	*Empty, no vial*
V13	~4.5‐mL absolute ethanol
V14	> 3‐mL 1‐M HCl
V15	Water bag spike with 100‐mL GE water bag
V16	~4.5‐mL sterile saline for injection (NS)
V17	42‐cm‐long silicone tubing connected to V5
V18	1/16″ PTFE tubing connected to Cyclotron/Process Panel CO_2_ output (purged with N_2_ gas for > 5 min)
V19	42‐cm‐long silicone tubing connected to the yellow 14‐cm‐long silicone tubing from the glass reactor via a F/F Luer adaptor
V20	*Empty*
V21	Waters C18 Plus Long Cartridge (800 mg), conditioned using 5‐mL absolute (200 proof) ethanol, then 10‐mL water, dried, and installed on cassette
V22	14‐cm‐long silicone tubing connected to C18 Plus Long Cartridge at V21
V23	*Empty*
V24	5 mL syringe (syringe #3)
V25	42‐cm‐long silicone tubing connect to dose vial with a 0.22 μm Cathivex‐GV filter
Grignard vial	10 mL vial purged with N_2_ gas for > 5 min then added 2 mL 0.1 M *n*‐PrMgCl (100 μL of 2 M *n*‐PrMgCl diluted with 1900 μL ether) (only added once prompted by timelist), connected to V8
LAH vial	2 mL vial purged with N_2_ gas for > 5 min then added 1 mL 0.3 M LAH (300 μL of 1 M LAH diluted with 700 μL ether), to be connected to V1 and V4 when prompted by timelist, clamped on a stand
Dose vial	10 mL sterile vial containing sterile saline for injection (5 mL)

*Note:* In green—Items to add when prompted by the FASTLab timelist. In orange—items that are on the FASTLab cassette that cannot be changed. In blue—items that are connected to the glass reactor.

Upon installation of the cassette and the completion of the initial cassette test, a prompt on the FASTLab ensures the installation of the inverted LAH vial between valve 1 (V1) and V4 (see Figure S2 for more details). Subsequently, a second prompt ensures the transfer of the Grignard solution, connected on V8, to the N_2_ purged reactor vial by vacuum. The door to the hot cell was closed once the FASTLab was ready to receive radioactivity. At the end‐of‐bombardment (EOB), [^11^C]CO_2_ was bubbled into the Grignard solution at a flow rate of 30 ± 5 mL/min for 4 min. Immediately afterwards, the LAH solution was transferred into the reaction mixture to reduce the intermediate to the final product (Scheme 1). The ether was evaporated at room temperature under a N_2_ stream at 100 mL/min for 5 min, and 1.5 mL of a 1 M HCl aqueous solution was added. The crude reaction mixture was diluted with 6 mL of water and pushed through a conditioned (5 mL absolute ethanol followed by 5 mL water) Waters C18 Plus Long cartridge. The reactor was rinsed with another 7 mL of water and eluted through the C18 Plus Long cartridge. The cartridge was then washed with an additional 14 mL of water to remove any impurities from the cartridge and dried for 15 s using N_2_ overpressure. [^11^C]Butanol was eluted off the C18 cartridge using 0.5 mL ethanol USP, followed by 4.5 mL 0.9% sodium chloride (normal saline) USP, through a Millipore Millex‐GS 0.22 μm filter into the sterile dose vial containing 5 mL normal saline. A sample was removed from the product vial and was submitted for quality control (QC) testing.

### 2.3 Radiosynthesis of [^11^C]Butanol on TracerLab FX Modules

**FIGURE 2:**
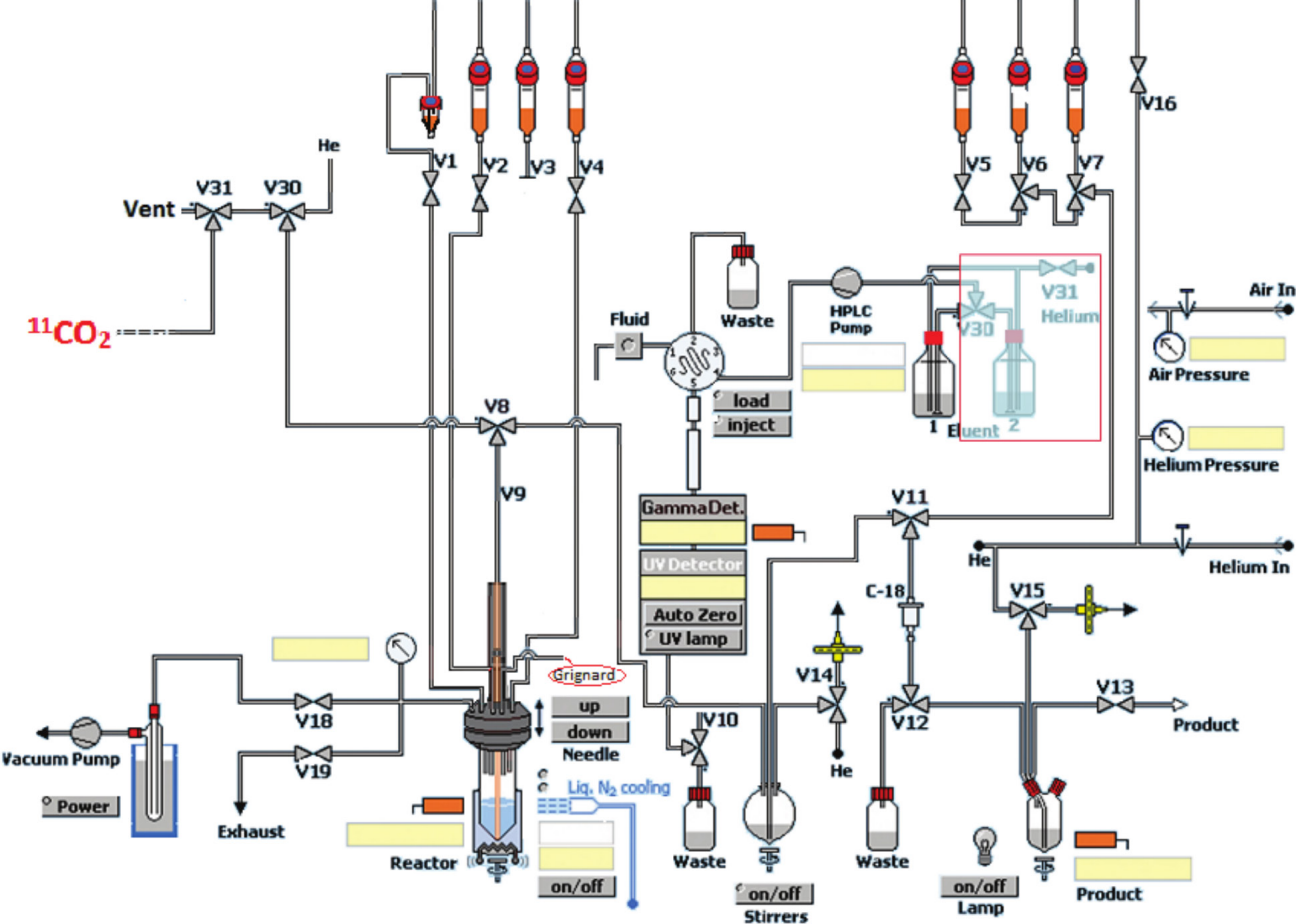
TracerLab FX_M_ module configuration for the synthesis of [^11^C]butanol. Highlighted in the red box is a modification reassigning V30 and V31 to control the addition of [^11^C]CO_2_. The HPLC portion of the FX_M_ and V3 are not used for this synthesis.

For comparison, [^11^C]butanol was also synthesized on either a TracerLab FX_M_ or FX_C‐Pro_ synthesis module through adaptation of previously reported methods [2, 11, 13]. The FX_M_ synthesis module was set up as depicted in Figure 2 and listed in Table 2. For synthesis on the FX_C‐Pro_ synthesizer, [^11^C]CO_2_ from the cyclotron was rerouted to the reactor through V27 (which normally is the exhaust), passing through the non‐operational CH_4_ trap. Briefly, 2 M *n*‐PrMgCl was diluted to a concentration of 0.1 M with ether and added to the N_2_ purged reactor 5 min prior to EOB. At EOB, [^11^C]CO_2_ was bubbled into the Grignard solution at a flow rate of 30 ± 5 mL/min for 4 min. LAH solution (0.15 M, diluted with ether) was then transferred into the reaction mixture. After 1 min, ether was evaporated under a stream of He at 150 ± 10 mL/min for 5 min at room temperature. Subsequently, 1.5 mL of 1 M HCl was added, and the crude reaction mixture was transferred to the dilution flask containing 10 mL water, followed by 5 mL water to rinse the reactor. This mixture was passed through a conditioned Waters C18 Plus Long cartridge to trap the product. The cartridge was washed with 5 mL water to remove any impurities and dried for 1 min with He gas. The final product [^11^C]butanol was eluted from the C18 cartridge using 0.5 mL ethanol USP, followed by 2.5 mL normal saline USP through a Millipore Millex‐GS 0.22 μm filter into the sterile final product vial containing 7 mL normal saline. A sample was removed from the final product vial and submitted for QC testing.

**TABLE 2:** TracerLab FX_M_ configuration and components.

Position/vial	Component
Reaction vessel	1 mL of 0.1 M *n*‐propyl magnesium chloride in either (50 μL of 2 M *n*‐PrMgCl diluted with 950 μL ether), added when prompted by the timelist
Vial 1	2 mL vial purged with N_2_ gas for > 5 min then added 1 mL of 0.15 M lithium aluminum hydride (150 μL of 1 M LAH diluted with 850 μL ether), connected to V1
Vial 2	1.5 mL of 1 M HCl
Vial 3	*Empty*
Vial 4	10 mL of water
Vial 5	5 mL of water
Vial 6	0.5 mL of absolute ethanol
Vial 7	2.5 mL of sterile saline for injection
Round‐bottom dilution flask	10 mL of water
Product vial	7 mL of sterile saline for injection

*Note:* In green—Items to add when prompted by the TracerLab FX_C‐Pro_ or FX_M_ timelist.

### 2.4 Quality Control Testing

**FIGURE 3:**
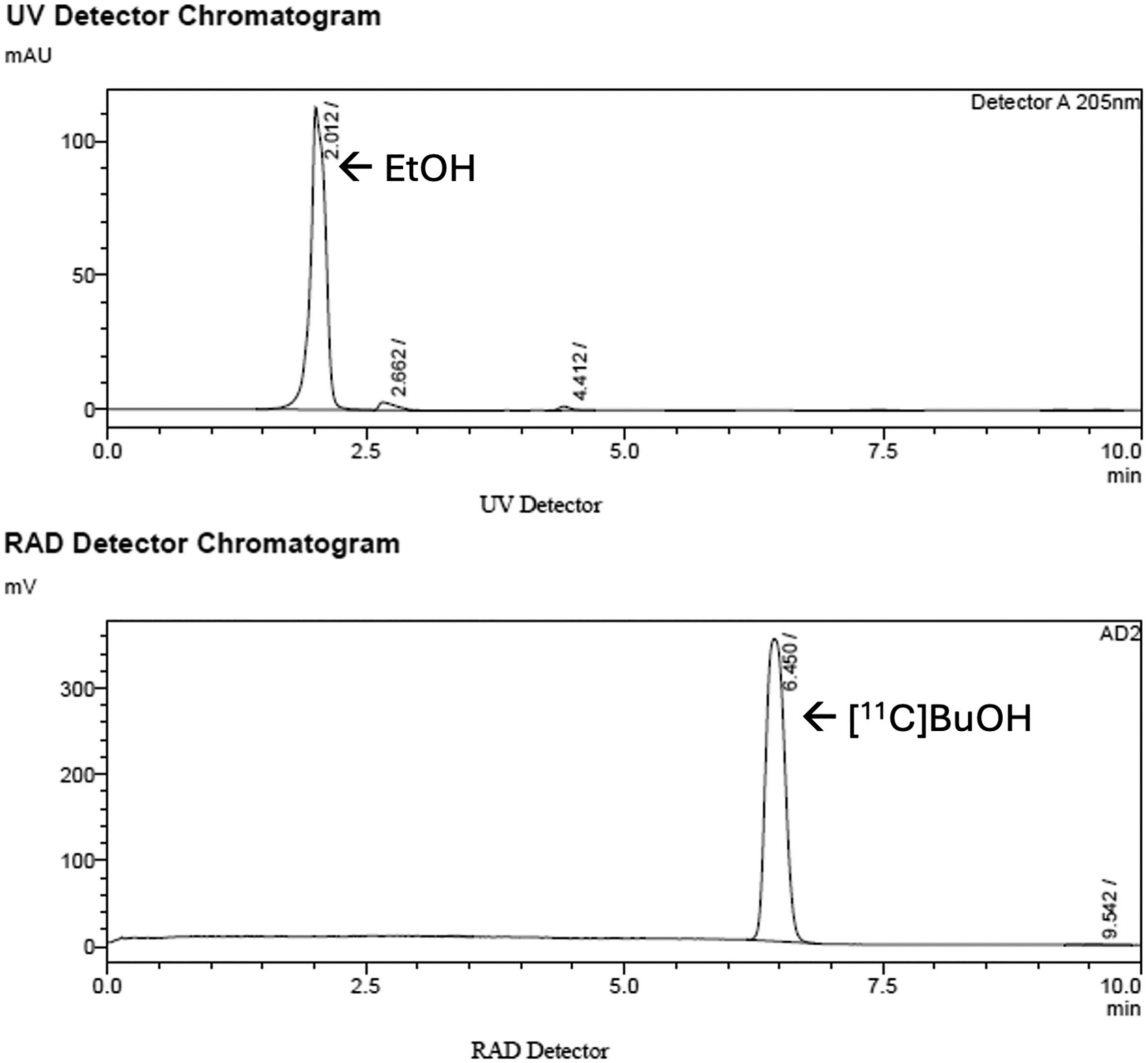
Representative analytical HPLC chromatograph for [^11^C]butanol obtained on Shimadzu LC2010A analytical equipment. Sample HPLC traces for [^11^C]butanol using the Luna C18(2) column method. Samples (10 μL) obtained from the FASTLab were diluted with 90 μL of 20% acetonitrile in the autosampler prior to injection. Minor peaks at 2.7 and 4.4 min were unidentified, but one could be MeCN used to dilute the sample.

[^11^C]Butanol was tested for appearance, pH, radiochemical purity (RCP) and residual solvents according to US Pharmacopeia chapter *<823>* and as previously reported [12, 14, 15]. Sterility and endotoxin were not completed as the workflow changes were upstream of the sterile filter, and all aseptic manipulations are comparable with published methods on the TracerLab that have previously passed endotoxin and sterility [12, 15]. A new HPLC quality control analysis system was developed with the following parameters: column: Phenomenex Luna C18(2), 5 μm, 100 Å, 250 × 4.6 mm; mobile phase: 20% acetonitrile; flow rate: 1.2 mL/min; oven: 40°C; UV detection at 205 nm. A representative chromatograph is shown in Figure 3 (t_R_ butanol ~6.5 min, limit of detection [LOD] = 2 mg/mL). Identity was confirmed via co‐injection of [^12^C]butanol reference standard (Figure S3). For comparison, a chromatograph of the previously reported HPLC method is shown in Figure S4 (t_R_ butanol ~ 12 min) [12, 14]. Residual solvent analysis was performed using undiluted samples to ensure residual ether was below the allowed limit. Since only class 3 solvents (ether and ethanol) are used in this synthesis, residual solvent testing is not required for routine batches [15, 16].

## 3 Results and Discussion

With the increasing demand in using [^11^C]butanol for CBF measurements, a robust and reproducible synthesis is required. An automated, cassette‐based synthesizer, like the FASTLab, can help improve consistency and reduce day‐of‐synthesis setup time, which, in turn, will increase the availability of [^11^C]butanol. Since [^11^C]butanol is prepared using a two‐step synthesis utilizing air‐sensitive Grignard and LAH reagents (Scheme 1), optimization of the reaction conditions is required. Our initial focus was on the optimization of the concentration of these two reagents. All experiments were carried out by bubbling [^11^C]CO_2_ into 1 mL of various concentrations of *n*‐PrMgCl in ether for 5 min, with subsequent quenching of the reaction with aqueous HCl. The trapped activities, in the form of [^11^C]butyric acid, are shown in Table 3. As expected, a higher concentration of Grignard reagent resulted in the trapping of increased amounts of [^11^C]CO_2_. However, precipitated salts from the higher Grignard concentration are expected, increasing the possibility of clogging lines when the reactions were performed using automated modules. To avoid excess precipitates, 0.1 M was selected as the Grignard reagent concentration for the production of [^11^C]butanol.

**TABLE 3:** Yields of [^11^C]butyric acid from various Grignard concentrations.

Grignard concentration (M)	0.05	0.1	0.2	0.4
Yields (mCi)	25	87	108	127
Radiochemical yield (%)	8.3	29	36	42.3

The efficiency of the reduction step was then studied using different volumes of 1 M LAH solution, ranging from 150 to 600 μL, and the results are summarized in Table 4. For all volumes of LAH tested, over 80% of the [^11^C]butyric acid was reduced to [^11^C]butanol in 5 min at room temperature, as indicated by HPLC analysis. A smaller volume of LAH (150 μL) was selected to minimize precipitation that could also lead to synthesis failures from clogged lines and considered as the practical lower limit for use in a fully automated system.

**TABLE 4:** Radiochemical conversion of [^11^C]butyric acid to [^11^C]butanol.

1M LAH volume (μL)	RCC of [^11^C]butyric acid (%)^a^	RCC of [^11^C]butanol (%)^a^
600	17	83
400	10	90
300	12 ± 4.2^b^	88 ± 4.2^b^
150	19 ± 1^b^	81 ± 1^b^

^a^ Radiochemical conversion (RCC), the fraction of radioactivity corresponding to the product in the crude reaction mixture as determined by HPLC.

^b^ *n* = 3.

Purification of [^11^C]butanol can be challenging because of its solubility. Due to its amphiphilic nature, [^11^C]butanol is only partially retained on commonly used reverse‐phase HPLC columns and SPE cartridges. Testing SPE cartridges with different sizes of hydrophobic resin allowed us to minimize impurity content while maximizing the amount of product in the final dose, as outlined in Table 5. Examination of various C18 resin bed sizes showed that the 360 mg SPE cartridge resulted in increased unretained [^11^C]butanol, while the use of a cartridge with greater than 2 g of resin resulted in excessive amounts of [^11^C]butanol stuck on the cartridge after elution. All three resins tested with approximately 1 g of C18 resin showed both good retention of the product after the initial loading and efficient elution with ethanol after rinsing the cartridge with water. The C18 Plus Long (820 mg) and C18 Vac (1 g) cartridges were also efficient, producing high yields of [^11^C]butanol with > 90% RCP. Since the C18 Vac syringe cartridge requires an extra adapter, the C18 Plus Long cartridge was chosen for routine production.

**TABLE 5:** [^11^C]Butanol retention and release from different SPE cartridges, based on activity.

Cartridges (Waters)	Unretained (%)	Washes (%)	Final dose (%)^a^	Retained on cartridge (%)
C18 plus short (360 mg)	66.1	12.3	19.6	2.0
C18 plus short (360 mg) × 3	29.9 ± 2.5^b^	17.9 ± 7.1^b^	42.5 ± 6.8^b^	9.8 ± 1.9^b^
C18 plus long (820 mg)	36.3 ± 1.1^b^	10.7 ± 1.7^b^	49.9 ± 1.8^b^	3.3 ± 1.2^b^
C18 6 cc Vac (1 g)	29.5 ± 0.7^b^	12.7 ± 0.7^b^	49.4 ± 3.5^b^	8.7 ± 2.2^b^
C18 12 cc Vac (2 g)	6.9	8.8	19.5	64.6

^a^ RCPs of all final doses were greater than 98%.

^b^ *n* = 3.

With optimized concentrations for the Grignard and LAH reagents identified, and an appropriate SPE cartridge selected, our focus shifted to automating the radiosynthesis of [^11^C]butanol on the FASTLab (Figure 1) and TracerLab FX (Figure 2) synthesis modules. Synthesis on the FASTLab required additional modifications of the cassette to accommodate workflow differences. These included (1) increasing the volume of Grignard reagent used (2 mL instead of 1 mL) and LAH used (300 μL instead of 150 μL in 1 mL ether) to compensate for transfer losses, reactor shape, and solvent evaporation, (2) changing from cyclin olefin copolymer (COC) reactors to glass reactors for chemical compatibility with ether, and (3) modifying the volume of wash solutions used during the purification step due to volume restrictions of the FASTLab. As summarized in Table 6, these modifications led to a non‐decay‐corrected RCY of 180 mCi (23% based upon 800 mCi of [^11^C]CO_2_ from a 60 μA, 5 min irradiation) and 99.4% RCP. Although the volumes of Grignard and LAH were increased, since the inner diameter of the FASTLab tubing is larger, and the LAH vial was inverted to remove precipitates, line clogging was not observed. Finally, to account for potential batch variations due to syringe measurement tolerances in synthesis and purification, an additional cartridge wash with water was added for the final FASTLab program.

**TABLE 6:** Optimization of procedure for synthesis on FASTLab.

Run	0.1 M Grignard volume	1M LAH volume	Yield (mCi)	Radiochemical yield (%)	Radiochemical purity (HPLC%)
1	1 mL	150 μL	0.81	0.1	8.4
2	2 mL	150 μL	71.2	8.9	70.1
3	2 mL	300 μL	179.8	22.5	99.4

To validate the process, this optimized FASTLab procedure was executed in triplicate, yielding 67–125 mCi [^11^C]butanol (4%–8% non‐decay corrected RCY, 10 min irradiation) after 21 min syntheses with RCPs > 95% (Table 7). Samples were submitted for solvent analysis by GC, indicating 5.6% ± 0.2% ethanol and 80.7 ± 9.3 μg/mL of ether, which was well below the 5000 μg/mL maximum allowed concentration for ether. The ethanol content is slightly elevated than the expected 5%, due to measuring tolerances in the syringe driver and dead volumes in the value actuators. The volume used to elute the SPE resin can be reduced to yield a total concentration of less than 5%. If desired, an ethanol‐free formulation with a preparative HPLC purification can be used, as discussed by Oyeniran and colleagues [13], but this will require accessorizing the FASTLab with HPLC+ [17].

**TABLE 7:** QC data for process verification batches of [^11^C]butanol using FASTLab.

QC test	Release criteria	Batch 1	Batch 2	Batch 3
Target irradiation current and time	—	60 μA × 10 min	60 μA × 10 min	60 μA × 10 min
Visual inspection	Clear, no ppt	Pass	Pass	Pass
pH	4.5–7.5	5.0	5.0	5.0
Radioactivity conc.	≥ 30 mCi/10 mL	125	92	67
Concentration	Reported Result (μg/mL)	< LOD^a^	< LOD^a^	< LOD^a^
Molar activity^b^	Reported result (Ci/mmol)	> 0.46	> 0.34	> 0.25
Radiochemical purity	≥ 90%	100	100	98
Radiochemical identity	RRT^c^ = 0.9–1.1	1.01	1.02	1.01

^a^ LOD = 2 mg/mL.

^b^ Molar activity assuming LOD; in reality, it is likely much higher.

^c^ RRT = relative retention time ([^11^C]butanol retention time/[^12^C]butanol retention time).

To compare the benefits of using the FASTLab to synthesize [^11^C]butanol compared to previously reported procedures, we also include the synthesis of [^11^C]butanol using a GE TracerLab synthesis module [12]. As previously reported, the GE TracerLab synthesis module and timelist were modified to make it compatible with the small volumes of reaction reagents used to prepare [^11^C]butanol (Figure 2). The modifications were the following: (1) The reactor was constantly purged with N_2_ or He to minimize atmospheric water; (2) the Grignard reagent was directly injected into the reactor under inert gas to prevent reaction with moisture and [^12^C]CO_2_ from air; (3) [^11^C]CO_2_ was delivered directly to the reactor from a molecular sieve column through a three‐way valve (which bypass the MeI system if a FX_C‐pro_ was used); and (4) a small volume of LAH was transferred from a sealed vial into the reactor. Using either TracerLab FX_M_ or FX_C‐Pro_ modules, the total synthesis time was 25 min from the end‐of‐bombardment. The RCY was 4%–7% based on [^11^C]CO_2_ (non‐decay corrected) and RCP was > 90% (Table 8). Samples that were submitted for solvent analysis by GC indicated 4.7% ethanol in the formulation, and residual solvents were below the allowable 5000 ppm limits for acetone (14 μg/mL) and ether (34 μg/mL). Acetone was tested as the synthesis of [^11^C]butanol on the TracerLab FX_M_ and FX_C‐Pro_ requires additional cleaning with ethanol and acetone to avoid cross‐contamination with tracers synthesized using the same modules (cf. single use cassettes employed with FASTLab).

**TABLE 8:** QC data for process verification batches of [^11^C]butanol using GE TracerLab FX modules.

QC test	Release criteria	Batch 1	Batch 2	Batch 3
TracerLab module	—	FX_C‐pro_	FX_C‐pro_	FX_M_
Target irradiation current and time	—	40 μA × 30 min	40 μA × 30 min	60 μA × 40 min
Visual inspection	Clear, no ppt	Pass	Pass	Pass
pH	4.5–7.5	5.5	5.0	5.0
Radioactivity conc.	≥ 30 mCi/10 mL	125	165	277
Concentration	Reported result (μg/mL)	< LOD^a^	< LOD^a^	< LOD^a^
Molar activity^b^	Reported result (Ci/mmol)	> 0.46	> 0.61	> 1.03
Radiochemical purity	≥ 90%	100	100	90
Radiochemical identity	RRT^c^ = 0.9–1.1	1.03	1.04	1.01

^a^ LOD = 2 mg/mL.

^b^ Molar activity assuming LOD; in reality, it is likely much higher.

^c^ RRT = relative retention time ([^11^C]butanol retention time/[^12^C]butanol retention time).

Similar to the FASTLab method, air sensitive reagents, Grignard and LAH, must be prepared and added 5 min prior to EOB to minimize reaction with air moisture. On the FASTLab method, the Grignard and LAH vials are prepared ahead of time and sealed, which reduces the day‐of‐synthesis setup time. Additionally, the synthesis on FASTLab is faster and does not require extensive cleaning between syntheses, further reducing the cost and improving operational simplicity. An initial cost comparison of the syntheses in the different modules revealed that although there is a cost associated with the FASTLab developer materials, it is less when accounting for the number of personnel hours required for setting up and executing a synthesis on the TracerLab synthesizer. Finally, the synthesis on FASTLab does not require module reconfiguration as required for the TracerLab FX_M_ and FX_C‐Pro_ syntheses, further decreasing workflow complexity and eliminating potential points of failure.

Lastly, when comparing HPLC quality control methods, the new method employing a Luna C18(2) column is preferred (compared to earlier reported methods) as, in our hands, Luna C18(2) is a more robust column than, for example, the Rezex RCM‐Monosaccharide Ca^+2^ column that we have used previously for [^11^C]butanol QC (see Supporting Information and [12] for more details). The Luna C18(2) column also leads to cleaner and easier to interpret UV chromatogram and shorter retention times, resulting in faster analysis which is favored for carbon‐11 radiopharmaceuticals.

## 4 Conclusions

The fully automated cassette‐based radiosynthesis of [^11^C]butanol on a GE FASTLab 2 was achieved in moderate yield and with excellent radiochemical purity. Through minimal adjustments to a prior TRACERLab synthesis, preparing [^11^C]butanol on the FASTLab allows for a simplified workflow. In particular, the single‐use cassette eliminates cleaning requirements for traditional fixed‐tube synthesizers to avoid cross‐contamination between tracers, leading to a more expedient day‐of‐synthesis setup and avoiding non‐standard module reconfiguration. Reflecting this, the optimized synthesis of [^11^C]butanol on the FASTLab 2 was comparable to the GE TracerLab FX modules in terms of product quality, but with a simpler and faster setup. Overall, these benefits are expected to offer high reproducibility and reliability of production, preparing us to meet the increased demand for [^11^C]butanol we are facing at our facility. Doses produced using the FASTLab are suitable for clinical applications.

## Conflicts of Interest

The authors declare no conflicts of interest. Given his role as an Academic Editor at JLCR, Peter J.H. Scott had no involvement in the peer review of this article and has no access to information regarding its peer review.

## Supporting information


**Figure S1:** Timelist for [^11^C]butanol on FASTLab 2.
**Figure S2:** Representative images for needle placement of the LAH vial when punctured and attached onto the FASTLab Cassette.
**Figure S3:** Representative analytical HPLC chromatographs using two analytical methods for [^11^C]butanol. Sample HPLC traces for [^11^C]butanol using the new Luna C18(2) column method.
**Figure S4:** Representative analytical HPLC chromatographs using the previously reported Rezex RCM‐monosaccharide Ca^+2^ column method.

## Data Availability

Raw data are included in the article and associated Supporting Information.
