# Supplementary material for: Fully Automated Radiosynthesis of No‐Carrier‐Added [11C]Butanol Using the GE FASTLab 2 Module
Source: J Labelled Comp Radiopharm. 2025 Jul 31;68(9-10):e4158. doi: 10.1002/jlcr.4158 (PMC12314111; doi:10.1002/jlcr.4158)
Supplement: Supplementary file 1 — Figure S1: Timelist for [11C]butanol on FASTLab 2. Figure S2: Representative images for needle placement of the LAH vial when punctured and attached onto the FASTLab Cassette. Figure S3: Representative analytical HPLC chromatographs using two analytical methods for [11C]butanol. Sample HPLC traces for [11C]butanol using the new Luna C18(2) column method. Figure S4: Representative analytical HPLC chromatographs using the previously reported Rezex RCM‐monosaccharide Ca+2 column method. [file JLCR-68-0-s001.pdf]

Fully Automated Radiosynthesis of No-Carrier-Added [^{11}C]butanol Using GE FASTLab 2 or TracerLab FX Modules

Ivan E. Wang[1], Jason A. Witek[2], Ryan J. Pakula[2], Bradford D. Henderson[2], Marianna Dakanali[2], Xia Shao[2*], Peter J.H. Scott[1,2,3*]

1. Department of Medicinal Chemistry, College of Pharmacy, University of Michigan, Ann Arbor, MI 48109, USA
2. Department of Radiology, University of Michigan, Ann Arbor MI, 48109, USA
3. Department of Pharmacology, University of Michigan, Ann Arbor MI, 48109, USA

*Corresponding Authors
Peter J. H. Scott and Xia Shao.
Department of Radiology, University of Michigan,
Ann Arbor, MI 48109, USA.
Email (PJHS): pjhscott@umich.edu
Email (XS): xshao@umich.edu

Supplemental Information

Figure S1: Timelist for [^{11}C]butanol on FASTLab 2……………………………………………………………………………..…… S2
Figure S2: Representitive images for needle placement of the LAH vial when punctured and attached onto the FASTLab Cassette… S6
Figure S3: Representative analytical HPLC chromatographs using two analytical methods for [^{11}C]butanol. Sample HPLC traces for [^{11}C]butanol using the new Luna C18(2) column method (a) and zoom of Fig 3a showing co-injected reference standard (b)..……..… S7
Figure S4: Representative analytical HPLC chromatographs using the previously reported Rezex RCM-monosaccharide Ca^{+2} column method ……………………………………………………………………………..……………………………………………...… S8

Figure S1: Timelist for [^{11}C]butanol on FASTLab 2.

The column headings from left to right and their corresponding parameter: columns description in *blue italics* are not used for this procedure.
White Columns:
1. Step number – defines the timelist order.
2. Message – description of the step

Yellow:
1. Duration of step (seconds)
2. Syringe #1 (1 mL) displacement (mm) at V3
3. Syringe #2 (5 mL) displacement (mm) at V11
4. Syringe #3 (5 mL) displacement (mm) at V24
5. Pressure settings (psi)
6. Vacuum settings (psi)
7. *Reactor 1 temperature*
8. *Reactor 2 temperature*

Pink:
1. *Reactor cooling using compressed air (1-on, 0-off)*
2. LF (low flow) nitrogen gas to cassette (1-on, 0-off)
3. HF (high flow) nitrogen gas to cassette (1-on, 0-off)
4. pressure reservoir (1-on, 0-off)
5. vacuum reservoir (1-on, 0-off)
6. *Vacuum 18O-water vial (1-on, 0-off)*
7. *Activity inlet (1-on, 0-off)*
8. Cassette exhaust (1-on, 0-off)
9. Waste repressure (1-on, 0-off)

Blue:
1. Valves 1 through 25 and their positions (C – center, L – left, R – right)

White:
1. *Controls 1-8 (allows hookup to multiple FASTLab or FASTLab HPLC+)*

Green:
1. Events (10-90, 100)
    a. Events with a 100-event code requires manual actions (e.g. adding an external vial) and a subsequent acknowledgement on the FASTLab timelist to proceed. These "event actions" are written in the "notes" column. The exact actions are listed below corresponding to each step with a 100-event code.
2. Default Condition
3. Custom Condition
4. Active Condition

White:
1. Report Parameters
2. Notes (see above, Events column)

Steps 1 through 55

Conditioning resins, installing LAH, adding Grignard, activity addition, reaction, and quenching.

Step	Message	Du…	Sy…	Syri…	Syr…	Pr…	Vacu…	R	R…	C…	LF…	H…	P…	V…	V…	A…	C…	Wa…	V1	V2	V3	V4	V5	V6	V7	V8	V9	V10	V11	V12	V13	V14	V15	V16	V17	V18	V19	V20	V21	V22	V23	V24	V25	E…	E…	Ext…	E…	E…	E…	E…	E…	Event	Default…	Cus…	Acti…	R…
1	Starting point	1	0	0	0	0	0	0	0	0	0	0	0	0	0	0	0	0	C	C	C	C	C	C	C	C	C	C	C	C	C	C	C	C	C	C	C	C	C	C	C	C	C	0	0	0	0	0	0	0	0	0	0	0	0	0
2	Connect a Luer Lock adaptor betwee…	1	0	0	0	0	0	0	0	0	0	0	0	0	0	0	0	0	C	C	C	C	C	C	C	C	C	C	C	C	C	C	C	C	C	C	C	C	C	C	C	C	C	0	0	0	0	0	0	0	0	100	0	0	0	0
3	Purge V1 through V4 (through adapt…	15	0	0	0	400	-400	0	0	0	0	1	0	0	0	0	1	0	L	C	C	R	C	C	C	C	C	C	C	C	C	C	C	C	C	C	C	C	C	C	C	C	C	0	0	0	0	0	0	0	0	0	2	-1	0	0
4	Flushing the manifold	5	0	0	0	800	-200	0	0	0	0	1	0	0	0	0	1	0	C	C	C	C	C	C	C	C	C	C	C	C	C	C	C	C	C	C	C	C	C	C	C	C	C	0	0	0	0	0	0	0	0	0	1	-1	0	0
5	NS vial at V16 pressurization	5	0	0	0	800	-200	0	0	0	0	1	0	0	0	0	0	0	C	C	C	C	C	C	C	C	C	C	C	C	C	C	C	L	C	C	C	C	C	C	C	C	C	0	0	0	0	0	0	0	0	0	0	0	0	0
6	NS vial at V16 pressurization	10	0	0	0	800	-200	0	0	0	0	1	0	0	0	0	1	0	C	C	C	C	C	C	C	C	C	C	C	C	C	C	C	C	C	C	C	C	C	C	C	C	C	0	0	0	0	0	0	0	0	0	0	0	0	0
7	Water Bag pressurization	3	0	0	0	200	-200	0	0	0	0	1	0	0	0	0	0	0	C	C	C	C	C	C	C	C	C	C	C	C	C	C	L	C	C	C	C	C	C	C	C	C	C	0	0	0	0	0	0	0	0	0	3	0	3	0
8	Water Bag pressurization	10	0	0	0	800	-200	0	0	0	0	1	0	0	0	0	1	0	C	C	C	C	C	C	C	C	C	C	C	C	C	C	C	C	C	C	C	C	C	C	C	C	C	0	0	0	0	0	0	0	0	0	3	0	3	0
9	1 M HCl vial pressurization	5	0	0	0	800	-200	0	0	0	0	1	0	0	0	0	0	0	C	C	C	C	C	C	C	C	C	C	C	C	C	L	C	C	C	C	C	C	C	C	C	C	C	0	0	0	0	0	0	0	0	0	0	0	0	0
10	1 M HCl vial pressurization	10	0	0	0	800	-200	0	0	0	0	1	0	0	0	0	1	0	C	C	C	C	C	C	C	C	C	C	C	C	C	C	C	C	C	C	C	C	C	C	C	C	C	0	0	0	0	0	0	0	0	0	0	0	0	0
11	EtOH vial pressurization	5	0	0	0	800	-200	0	0	0	0	1	0	0	0	0	0	0	C	C	C	C	C	C	C	C	C	C	C	C	L	C	C	C	C	C	C	C	C	C	C	C	C	0	0	0	0	0	0	0	0	0	0	0	0	0
12	EtOH vial pressurization	10	0	0	0	800	-200	0	0	0	0	1	0	0	0	0	1	0	C	C	C	C	C	C	C	C	C	C	C	C	C	C	C	C	C	C	C	C	C	C	C	C	C	0	0	0	0	0	0	0	0	0	0	0	0	0
13	EtOH vial pressurization	5	0	0	0	800	-200	0	0	0	0	1	0	0	0	0	0	0	C	C	C	C	C	C	C	C	C	C	C	L	C	C	C	C	C	C	C	C	C	C	C	C	C	0	0	0	0	0	0	0	0	0	0	0	0	0
14	Pull EtOH into Syringe 24	30	0	0	0	800	-1000	0	0	0	0	1	0	0	0	0	1	0	C	C	C	C	C	C	C	C	C	C	C	R	C	C	C	C	C	C	C	C	L	R	C	C	C	0	0	0	0	0	0	0	0	100	2	-1	0	0
15	Pull EtOH into Syringe 24	3	0	0	0	800	-200	0	0	0	0	0	0	0	0	0	1	0	C	C	C	C	C	C	C	C	C	C	C	C	C	C	C	C	C	C	C	C	C	C	C	R	C	0	0	0	0	0	0	0	0	0	1	-1	0	0
16	Pull up Water into Syringe 24	3	0	0	0	800	-200	0	0	0	0	1	0	0	0	0	0	0	C	C	C	C	C	C	C	C	C	C	C	C	C	C	R	C	C	C	C	C	L	R	C	L	C	0	0	0	0	0	0	0	0	0	0	0	0	0
17	Pull Water into Syringe 24	12	0	0	500	800	-200	0	0	0	0	0	0	0	0	0	0	0	C	C	C	C	C	C	C	C	C	C	C	C	C	C	R	C	C	C	C	C	L	R	C	L	C	0	0	0	0	0	0	0	0	0	0	0	0	0
18	Pull Water into Syringe 24	10	0	0	600	800	-200	0	0	0	0	1	0	0	0	0	0	0	C	C	C	C	C	C	C	C	C	C	C	C	C	C	C	C	C	C	C	C	L	R	C	L	C	0	0	0	0	0	0	0	0	0	0	0	0	0
19	Pull Water into Syringe 24	3	0	0	600	800	-200	0	0	0	0	0	0	0	0	0	1	0	C	C	C	C	C	C	C	C	C	C	C	C	C	C	C	C	C	C	C	C	C	C	C	R	C	0	0	0	0	0	0	0	0	0	0	0	0	0
20	Pull Water into Syringe 24	10	0	0	0	800	-200	0	0	0	0	0	0	0	0	0	1	0	C	C	C	C	C	C	C	C	C	C	C	C	C	C	C	C	C	C	C	C	C	C	C	R	C	0	0	0	0	0	0	0	0	0	0	0	0	0
21	Pull Water into Syringe 24	12	0	0	500	800	-200	0	0	0	0	0	0	0	0	0	0	0	C	C	C	C	C	C	C	C	C	C	C	C	C	C	R	C	C	C	C	C	L	R	C	L	C	0	0	0	0	0	0	0	0	0	0	0	0	0
22	Pull Water into Syringe 24	10	0	0	600	800	-200	0	0	0	0	1	0	0	0	0	0	0	C	C	C	C	C	C	C	C	C	C	C	C	C	C	C	C	C	C	C	C	L	R	C	L	C	0	0	0	0	0	0	0	0	0	0	0	0	0
23	Pull Water into Syringe 24	3	0	0	600	800	-200	0	0	0	0	0	0	0	0	0	1	0	C	C	C	C	C	C	C	C	C	C	C	C	C	C	C	C	C	C	C	C	C	C	C	R	C	0	0	0	0	0	0	0	0	0	0	0	0	0
24	Pull Water into Syringe 24	10	0	0	0	800	-200	0	0	0	0	0	0	0	0	0	1	0	C	C	C	C	C	C	C	C	C	C	C	C	C	C	C	C	C	C	C	C	C	C	C	R	C	0	0	0	0	0	0	0	0	0	0	0	0	0
25	Pull up Air into Syringe 24	3	0	0	0	800	-200	0	0	0	0	1	0	0	0	0	0	0	C	C	C	C	C	C	C	C	C	C	C	C	C	C	C	C	C	C	C	C	L	R	C	L	C	0	0	0	0	0	0	0	0	0	0	0	0	0
26	Pull Air into Syringe 24	12	0	0	600	800	-200	0	0	0	0	1	0	0	0	0	0	0	C	C	C	C	C	C	C	C	C	C	C	C	C	C	C	C	C	C	C	C	L	R	C	L	C	0	0	0	0	0	0	0	0	0	0	0	0	0
27	Pull Air into Syringe 24	3	0	0	600	800	-200	0	0	0	0	0	0	0	0	0	1	0	C	C	C	C	C	C	C	C	C	C	C	C	C	C	C	C	C	C	C	C	C	C	C	R	C	0	0	0	0	0	0	0	0	0	0	0	0	0
28	Pull Air into Syringe 24	3	0	0	0	800	-200	0	0	0	0	0	0	0	0	0	1	0	C	C	C	C	C	C	C	C	C	C	C	C	C	C	C	C	C	C	C	C	C	C	C	R	C	0	0	0	0	0	0	0	0	0	0	0	0	0
29	Dry resin	10	0	0	0	800	-200	0	0	0	0	1	0	0	0	0	1	0	C	C	C	C	C	C	C	C	C	C	C	C	C	C	C	C	C	C	C	C	L	R	C	C	C	0	0	0	0	0	0	0	0	0	0	0	0	0
30	Flushing manifold	15	0	0	0	10…	-200	0	0	0	0	1	0	0	0	0	1	0	C	C	C	C	C	C	C	C	C	C	C	C	C	C	C	C	C	C	C	C	C	C	C	C	C	0	0	0	0	0	0	0	0	0	3	0	3	0
31	Flushing V1 through V4 with nitrogen	15	0	0	0	800	-200	0	0	0	0	1	0	0	0	0	1	0	L	C	C	R	C	C	C	C	C	C	C	C	C	C	C	C	C	C	C	C	C	C	C	C	C	0	0	0	0	0	0	0	0	0	3	0	3	0
32	Flushing reactor with Nitrogen	60	0	0	0	800	-400	0	0	0	0	1	0	0	0	0	1	0	C	C	C	C	C	C	L	C	C	R	C	C	C	C	C	C	C	C	C	C	C	C	C	C	C	0	0	0	0	0	0	0	0	0	2	-1	0	0
33	Waiting to begin adding Grignard Re…	1	0	0	0	200	-50	0	0	0	0	1	0	0	0	0	1	0	C	C	C	C	C	C	L	C	C	R	C	C	C	C	C	C	C	C	C	C	C	C	C	C	C	0	0	0	0	0	0	0	0	100	1	-1	0	0
34	Adding Grignard Reagent	5	0	0	0	50	-200	0	0	0	0	0	0	0	0	0	1	0	C	C	C	C	C	C	R	L	C	R	C	C	C	C	C	C	C	C	C	C	C	C	C	C	C	0	0	0	0	0	0	0	0	0	2	-1	0	0
35	Waiting to END adding Grignard Re…	1	0	0	0	20	-200	0	0	0	0	0	0	0	0	0	1	0	C	C	C	C	C	C	R	L	C	R	C	C	C	C	C	C	C	C	C	C	C	C	C	C	C	0	0	0	0	0	0	0	0	100	3	-1	0	0
36	Flushing reactor with Nitrogen	10	0	0	0	40	-50	0	0	0	0	1	0	0	0	0	1	0	C	C	C	C	C	C	L	C	C	R	C	C	C	C	C	C	C	C	C	C	C	C	C	C	C	0	0	0	0	0	0	0	0	0	1	-1	0	0
37	Connecting LAH to V4 and V1 to Vent	1	0	0	0	0	0	0	0	0	0	0	0	0	0	0	0	0	C	C	C	C	C	C	C	C	C	C	C	C	C	C	C	C	C	C	C	C	C	C	C	C	C	0	0	0	0	0	0	0	0	100	1	-1	0	0
38	Buffer Step for Actiivty	1	0	0	0	0	0	0	0	0	0	0	0	0	0	0	0	0	C	C	C	C	C	C	C	C	C	C	C	C	C	C	C	C	C	C	C	C	C	C	C	C	C	0	0	0	0	0	0	0	0	0	0	-1	0	0
39	Purge Reactor with N2	120	0	0	0	20	-50	0	0	0	1	0	0	0	0	0	1	0	C	C	C	C	C	C	L	C	C	R	C	C	C	C	C	C	C	C	C	C	C	C	C	C	C	0	0	0	0	0	0	0	0	0	2	-1	0	0
40	Transfering CO2 from HC-3/4	1	0	0	0	200	0	0	0	0	0	0	0	0	0	0	1	0	C	C	C	C	C	C	C	C	C	C	C	C	C	C	C	C	C	C	C	C	C	C	C	C	C	0	0	0	0	0	0	0	0	10	1	-1	0	0
41	Transfering CO2 from HC-3/4	1	0	0	0	200	0	0	0	0	0	0	0	0	0	0	1	0	C	C	C	C	C	C	C	C	R	L	C	C	C	C	C	C	C	R	L	C	C	C	C	C	C	0	0	0	0	0	0	0	0	20	0	-1	0	0
42	Transfering CO2 from HC-3/4	1	0	0	0	200	0	0	0	0	0	0	0	0	0	0	1	0	C	C	C	C	C	C	C	C	R	L	C	C	C	C	C	C	C	R	L	C	C	C	C	C	C	0	0	0	0	0	0	0	0	30	0	-1	0	0
43	Transfering CO2 from HC-3/4	1	0	0	0	200	0	0	0	0	0	0	0	0	0	0	1	0	C	C	C	C	C	C	C	C	R	L	C	C	C	C	C	C	C	R	L	C	C	C	C	C	C	0	0	0	0	0	0	0	0	40	0	-1	0	0
44	Transfering CO2 from HC-3/4	1	0	0	0	200	0	0	0	0	0	0	0	0	0	0	1	0	C	C	C	C	C	C	C	C	R	L	C	C	C	C	C	C	C	R	L	C	C	C	C	C	C	0	0	0	0	0	0	0	0	50	0	-1	0	0
45	Transfering CO2 from HC-3/4	1	0	0	0	200	0	0	0	0	0	0	0	0	0	0	1	0	C	C	C	C	C	C	C	C	R	L	C	C	C	C	C	C	C	R	L	C	C	C	C	C	C	0	0	0	0	0	0	0	0	60	0	-1	0	0
46	Transfering CO2 from HC-3/4 (5 min)	300	0	0	0	200	0	0	0	0	0	0	0	0	0	0	1	0	C	C	C	C	C	C	C	C	R	L	C	C	C	C	C	C	C	R	L	C	C	C	C	C	C	0	0	0	0	0	0	0	0	0	0	-1	0	1
47	Dry Line	5	0	0	0	800	-200	0	0	0	0	1	0	0	0	0	1	0	C	C	C	C	C	C	C	C	C	C	C	C	C	C	C	C	C	C	C	C	C	C	C	C	C	0	0	0	0	0	0	0	0	0	2	-1	0	2
48	Dry Bypass	5	0	0	0	800	-200	0	0	0	0	1	0	0	0	0	1	0	C	C	C	C	L	C	C	C	C	C	C	C	C	C	C	C	R	C	C	C	C	C	C	C	C	0	0	0	0	0	0	0	0	0	0	-1	0	0
49	Add LAH into reactor	1	0	0	0	200	-1000	0	0	0	0	1	0	0	0	0	1	0	L	C	C	R	C	C	L	C	C	R	C	C	C	C	C	C	C	C	C	C	C	C	C	C	C	0	0	0	0	0	0	0	0	100	2	-1	0	0
50	Add LAH into reactor 2	5	0	0	0	800	-1000	0	0	0	0	1	0	0	0	0	1	0	L	C	C	R	C	C	L	C	C	R	C	C	C	C	C	C	C	C	C	C	C	C	C	C	C	0	0	0	0	0	0	0	0	0	3	-1	0	0
51	Stir and Evap	60	0	0	0	50	-200	0	0	0	1	0	0	0	0	0	1	0	C	C	C	C	L	C	C	C	R	L	C	C	C	C	C	C	R	C	L	C	C	C	C	C	C	0	0	0	0	0	0	0	0	0	1	-1	0	0
52	Evap 5 min	300	0	0	0	500	-200	0	0	0	1	0	0	0	0	0	1	0	C	C	C	C	L	C	C	C	R	L	C	C	C	C	C	C	R	C	L	C	C	C	C	C	C	0	0	0	0	0	0	0	0	0	3	-1	0	0
53	Pull up 1 M HCl to quench Reaction	3	0	30	0	800	-200	0	0	0	0	0	0	0	0	0	1	0	C	C	C	C	C	C	C	C	C	C	R	C	C	C	C	C	C	C	C	C	C	C	C	C	C	0	0	0	0	0	0	0	0	0	3	-1	0	0
54	Pull up 1 M HCl to quench Reaction	5	0	200	0	800	-200	0	0	0	0	0	0	0	0	0	1	0	C	C	C	C	R	C	C	C	L	C	R	C	C	L	C	C	R	L	C	C	C	C	C	C	C	0	0	0	0	0	0	0	0	0	0	-1	0	0
55	Pull up 1 M HCl to quench Reaction	3	0	200	0	800	-100	0	0	0	0	0	0	0	0	0	1	0	C	C	C	C	R	C	L	C	C	R	L	C	C	C	C	C	R	C	C	C	C	C	C	C	C	0	0	0	0	0	0	0	0	0	1	-1	0	0

Step 2 – Connect a Luer Lock adaptor between V1 and V4 then Continue

Step 14 – Continue once EtOH vial at V12 is empty

Step 33 – Waiting to BEGIN adding Grignard Reagent

Step 35 – Waiting to END adding Grignard Reagent via Vacuum
Step 37 – Connect Inverted LAH vial to V4 line then V1 to vent needle. Continue
Step 49 – Add LAH, ensure vial is empty then Continue.
Step 52 – NOTE: A low flow nitrogen setting of 500 is equivalent to 100 mL/min flow rate.

Steps 56 through 109

Quenching reaction, purification on C18 Plus Long, and washes

56	Pull up 1 M HCl to quench Reaction	3	0	75	0	800	-100	0	0	0	0	0	0	0	0	0	1	0	C	C	C	C	R	C	L	C	C	R	L	C	C	C	C	C	R	C	C	C	C	C	C	C	C	0	0	0	0	0	0	0	0	0	0	-1	0	0
57	Pull up 1 M HCl to quench Reaction	3	0	0	0	800	-100	0	0	0	0	0	0	0	0	0	1	0	C	C	C	C	R	C	L	C	C	R	L	C	C	C	C	C	R	C	C	C	C	C	C	C	C	0	0	0	0	0	0	0	0	0	0	-1	0	0
58	Pull up 1 M HCl to quench Reaction	3	0	0	30	800	-200	0	0	0	0	0	0	0	0	0	1	0	C	C	C	C	C	C	C	C	C	C	C	C	C	C	C	C	C	C	C	C	C	C	C	R	C	0	0	0	0	0	0	0	0	0	2	-1	0	3
59	Pull up Water to dilute Reaction	3	0	0	30	800	-200	0	0	0	0	0	0	0	0	0	1	0	C	C	C	C	C	C	C	C	C	C	C	C	C	C	R	C	C	C	C	C	C	C	C	L	C	0	0	0	0	0	0	0	0	0	0	-1	0	0
60	Pull up Water to dilute Reaction	3	0	0	413	800	-200	0	0	0	0	0	0	0	0	0	1	0	C	C	C	C	C	C	C	C	C	C	C	C	C	C	R	C	C	C	C	C	C	C	C	L	C	0	0	0	0	0	0	0	0	0	0	-1	0	0
61	Pull water	7	0	247	413	50	0	0	0	0	0	0	0	0	0	0	1	0	C	C	C	C	C	C	C	C	C	C	R	C	C	C	L	C	C	C	C	C	C	C	C	C	C	0	0	0	0	0	0	0	0	0	1	-1	0	4
62	Pull up Water Elution to Reactor	5	0	100	413	800	-200	0	0	0	0	0	0	0	0	0	1	0	C	C	C	C	C	C	C	C	C	C	R	C	C	C	C	C	C	C	L	C	C	C	C	C	C	0	0	0	0	0	0	0	0	0	2	-1	0	5
63	Pull up Water Elution to Reactor	5	0	500	413	800	-200	0	0	0	0	0	0	0	0	0	1	0	C	C	C	C	C	C	C	C	C	C	R	C	C	C	C	C	C	C	L	C	C	C	C	C	C	0	0	0	0	0	0	0	0	0	0	-1	0	6
64	Pull up Water Elution to Reactor	5	0	100	413	800	-200	0	0	0	0	0	0	0	0	0	1	0	C	C	C	C	C	C	C	C	C	C	R	C	C	C	C	C	C	C	L	C	C	C	C	C	C	0	0	0	0	0	0	0	0	0	0	-1	0	7
65	Pull up Water Elution to Reactor	10	0	500	413	800	-200	0	0	0	0	0	0	0	0	0	1	0	C	C	C	C	C	C	C	C	C	C	R	C	C	C	C	C	C	C	L	C	C	C	C	C	C	0	0	0	0	0	0	0	0	0	0	-1	0	8
66	Further Dilute reaction	10	0	600	200	800	-200	0	0	0	0	0	0	0	0	0	1	0	C	C	C	C	C	C	C	C	C	C	R	C	C	C	C	C	C	C	C	C	C	C	C	L	C	0	0	0	0	0	0	0	0	0	0	-1	0	0
67	Load C18 Plus Long	3	0	600	200	800	-200	0	0	0	0	0	0	0	0	0	1	0	C	C	C	C	C	C	C	C	C	C	R	C	C	C	C	C	C	C	C	C	L	R	C	C	C	0	0	0	0	0	0	0	0	0	0	-1	0	0
68	Load C18 Plus Long	3	0	550	200	800	-200	0	0	0	0	0	0	0	0	0	1	0	C	C	C	C	C	C	C	C	C	C	R	C	C	C	C	C	C	C	C	C	L	R	C	C	C	0	0	0	0	0	0	0	0	0	0	-1	0	0
69	Load C18 Plus Long	3	0	500	200	800	-200	0	0	0	0	0	0	0	0	0	1	0	C	C	C	C	C	C	C	C	C	C	R	C	C	C	C	C	C	C	C	C	L	R	C	C	C	0	0	0	0	0	0	0	0	0	0	-1	0	0
70	Load C18 Plus Long	3	0	450	200	800	-200	0	0	0	0	0	0	0	0	0	1	0	C	C	C	C	C	C	C	C	C	C	R	C	C	C	C	C	C	C	C	C	L	R	C	C	C	0	0	0	0	0	0	0	0	0	0	-1	0	0
71	Load C18 Plus Long	3	0	400	200	800	-200	0	0	0	0	0	0	0	0	0	1	0	C	C	C	C	C	C	C	C	C	C	R	C	C	C	C	C	C	C	C	C	L	R	C	C	C	0	0	0	0	0	0	0	0	0	0	-1	0	0
72	Load C18 Plus Long	3	0	350	200	800	-200	0	0	0	0	0	0	0	0	0	1	0	C	C	C	C	C	C	C	C	C	C	R	C	C	C	C	C	C	C	C	C	L	R	C	C	C	0	0	0	0	0	0	0	0	0	0	-1	0	0
73	Load C18 Plus Long	3	0	300	200	800	-200	0	0	0	0	0	0	0	0	0	1	0	C	C	C	C	C	C	C	C	C	C	R	C	C	C	C	C	C	C	C	C	L	R	C	C	C	0	0	0	0	0	0	0	0	0	0	-1	0	0
74	Load C18 Plus Long	3	0	250	200	800	-200	0	0	0	0	0	0	0	0	0	1	0	C	C	C	C	C	C	C	C	C	C	R	C	C	C	C	C	C	C	C	C	L	R	C	C	C	0	0	0	0	0	0	0	0	0	0	-1	0	0
75	Load C18 Plus Long	3	0	200	200	800	-200	0	0	0	0	0	0	0	0	0	1	0	C	C	C	C	C	C	C	C	C	C	R	C	C	C	C	C	C	C	C	C	L	R	C	C	C	0	0	0	0	0	0	0	0	0	0	-1	0	0
76	Load C18 Plus Long	3	0	150	200	800	-200	0	0	0	0	0	0	0	0	0	1	0	C	C	C	C	C	C	C	C	C	C	R	C	C	C	C	C	C	C	C	C	L	R	C	C	C	0	0	0	0	0	0	0	0	0	0	-1	0	0
77	Load C18 Plus Long	3	0	100	200	800	-200	0	0	0	0	0	0	0	0	0	1	0	C	C	C	C	C	C	C	C	C	C	R	C	C	C	C	C	C	C	C	C	L	R	C	C	C	0	0	0	0	0	0	0	0	0	0	-1	0	0
78	Load C18 Plus Long	3	0	50	200	800	-200	0	0	0	0	0	0	0	0	0	1	0	C	C	C	C	C	C	C	C	C	C	R	C	C	C	C	C	C	C	C	C	L	R	C	C	C	0	0	0	0	0	0	0	0	0	0	-1	0	0
79	Load C18 Plus Long	3	0	0	200	800	-200	0	0	0	0	0	0	0	0	0	1	0	C	C	C	C	C	C	C	C	C	C	R	C	C	C	C	C	C	C	C	C	L	R	C	C	C	0	0	0	0	0	0	0	0	0	0	-1	0	0
80	Dry C18 Plus Long	3	0	0	200	400	-200	0	0	0	0	1	0	0	0	0	1	0	C	C	C	C	C	C	C	C	C	C	C	C	C	C	C	C	C	C	C	C	L	R	C	C	C	0	0	0	0	0	0	0	0	0	3	-1	0	0
81	Further Dilute reaction	10	0	0	0	800	-200	0	0	0	0	0	0	0	0	0	1	0	C	C	C	C	C	C	C	C	R	L	R	C	C	C	C	C	C	C	R	C	C	C	C	L	C	0	0	0	0	0	0	0	0	0	3	-1	0	9
82	Further Dilute reaction	3	0	0	0	200	-200	0	0	0	0	1	0	0	0	0	1	0	C	C	C	C	C	C	C	C	C	L	R	C	C	C	C	C	C	C	L	C	C	C	C	R	C	0	0	0	0	0	0	0	0	0	3	-1	0	0
83	Further Dilute reaction	10	0	600	0	200	-200	0	0	0	0	1	0	0	0	0	1	0	C	C	C	C	C	C	C	C	C	L	R	C	C	C	C	C	C	C	L	C	C	C	C	R	C	0	0	0	0	0	0	0	0	0	0	-1	0	0
84	Further Dilute reaction	3	0	550	0	200	-200	0	0	0	0	0	0	0	0	0	1	0	C	C	C	C	C	C	C	C	C	C	R	C	C	C	C	C	C	C	C	C	L	R	C	C	C	0	0	0	0	0	0	0	0	0	0	-1	0	0
85	Further Dilute reaction	3	0	500	0	200	-200	0	0	0	0	0	0	0	0	0	1	0	C	C	C	C	C	C	C	C	C	C	R	C	C	C	C	C	C	C	C	C	L	R	C	C	C	0	0	0	0	0	0	0	0	0	0	-1	0	0
86	Further Dilute reaction	3	0	450	0	200	-200	0	0	0	0	0	0	0	0	0	1	0	C	C	C	C	C	C	C	C	C	C	R	C	C	C	C	C	C	C	C	C	L	R	C	C	C	0	0	0	0	0	0	0	0	0	0	-1	0	0
87	Further Dilute reaction	3	0	400	0	200	-200	0	0	0	0	0	0	0	0	0	1	0	C	C	C	C	C	C	C	C	C	C	R	C	C	C	C	C	C	C	C	C	L	R	C	C	C	0	0	0	0	0	0	0	0	0	0	-1	0	0
88	Load C18 Plus Long	3	0	350	0	800	-200	0	0	0	0	0	0	0	0	0	1	0	C	C	C	C	C	C	C	C	C	C	R	C	C	C	C	C	C	C	C	C	L	R	C	C	C	0	0	0	0	0	0	0	0	0	3	-1	0	0
89	Load C18 Plus Long	3	0	300	0	800	-200	0	0	0	0	0	0	0	0	0	1	0	C	C	C	C	C	C	C	C	C	C	R	C	C	C	C	C	C	C	C	C	L	R	C	C	C	0	0	0	0	0	0	0	0	0	0	-1	0	0
90	Load C18 Plus Long	3	0	250	0	800	-200	0	0	0	0	0	0	0	0	0	1	0	C	C	C	C	C	C	C	C	C	C	R	C	C	C	C	C	C	C	C	C	L	R	C	C	C	0	0	0	0	0	0	0	0	0	0	-1	0	0
91	Load C18 Plus Long	3	0	200	0	800	-200	0	0	0	0	0	0	0	0	0	1	0	C	C	C	C	C	C	C	C	C	C	R	C	C	C	C	C	C	C	C	C	L	R	C	C	C	0	0	0	0	0	0	0	0	0	0	-1	0	0
92	Load C18 Plus Long	3	0	150	0	800	-200	0	0	0	0	0	0	0	0	0	1	0	C	C	C	C	C	C	C	C	C	C	R	C	C	C	C	C	C	C	C	C	L	R	C	C	C	0	0	0	0	0	0	0	0	0	0	-1	0	0
93	Load C18 Plus Long	3	0	100	0	800	-200	0	0	0	0	0	0	0	0	0	1	0	C	C	C	C	C	C	C	C	C	C	R	C	C	C	C	C	C	C	C	C	L	R	C	C	C	0	0	0	0	0	0	0	0	0	0	-1	0	0
94	Load C18 Plus Long	3	0	50	0	800	-200	0	0	0	0	0	0	0	0	0	1	0	C	C	C	C	C	C	C	C	C	C	R	C	C	C	C	C	C	C	C	C	L	R	C	C	C	0	0	0	0	0	0	0	0	0	0	-1	0	0
95	Load C18 Plus Long	3	0	0	0	800	-200	0	0	0	0	0	0	0	0	0	1	0	C	C	C	C	C	C	C	C	C	C	R	C	C	C	C	C	C	C	C	C	L	R	C	C	C	0	0	0	0	0	0	0	0	0	0	-1	0	0
96	Dry C18 Plus Long	10	0	0	0	400	-200	0	0	0	0	1	0	0	0	0	1	0	C	C	C	C	C	C	C	C	C	C	C	C	C	C	C	C	C	C	C	C	L	R	C	C	C	0	0	0	0	0	0	0	0	0	3	-1	0	0
97	Pull up H2O to wash C18	3	0	30	0	800	-200	0	0	0	0	0	0	0	0	0	1	0	C	C	C	C	C	C	C	C	C	C	R	C	C	C	C	C	C	C	C	C	C	C	C	C	C	0	0	0	0	0	0	0	0	0	3	-1	0	0
98	Pull up H2O to wash C18	7	0	600	0	800	-200	0	0	0	0	0	0	0	0	0	1	0	C	C	C	C	C	C	C	C	C	C	R	C	C	C	L	C	C	C	C	C	C	C	C	C	C	0	0	0	0	0	0	0	0	0	0	-1	0	0
99	Pull up H2O to wash C18	3	0	600	0	800	-200	0	0	0	0	0	0	0	0	0	1	0	C	C	C	C	C	C	C	C	C	C	R	C	C	C	C	C	C	C	C	C	L	R	C	C	C	0	0	0	0	0	0	0	0	0	0	-1	0	0
100	Pull up H2O to wash C18	3	0	400	0	800	-200	0	0	0	0	0	0	0	0	0	1	0	C	C	C	C	C	C	C	C	C	C	R	C	C	C	C	C	C	C	C	C	L	R	C	C	C	0	0	0	0	0	0	0	0	0	0	-1	0	0
101	Pull up H2O to wash C18	3	0	200	0	800	-200	0	0	0	0	0	0	0	0	0	1	0	C	C	C	C	C	C	C	C	C	C	R	C	C	C	C	C	C	C	C	C	L	R	C	C	C	0	0	0	0	0	0	0	0	0	0	-1	0	0
102	Pull up H2O to wash C18	3	0	0	0	800	-200	0	0	0	0	0	0	0	0	0	1	0	C	C	C	C	C	C	C	C	C	C	R	C	C	C	C	C	C	C	C	C	L	R	C	C	C	0	0	0	0	0	0	0	0	0	0	-1	0	0
103	Pull up H2O to wash C18	3	0	30	0	800	-200	0	0	0	0	0	0	0	0	0	1	0	C	C	C	C	C	C	C	C	C	C	R	C	C	C	C	C	C	C	C	C	C	C	C	C	C	0	0	0	0	0	0	0	0	0	0	-1	0	0
104	Pull up H2O to wash C18	7	0	600	0	800	-200	0	0	0	0	0	0	0	0	0	1	0	C	C	C	C	C	C	C	C	C	C	R	C	C	C	L	C	C	C	C	C	C	C	C	C	C	0	0	0	0	0	0	0	0	0	0	-1	0	0
105	Pull up H2O to wash C18	3	0	600	0	800	-200	0	0	0	0	0	0	0	0	0	1	0	C	C	C	C	C	C	C	C	C	C	R	C	C	C	C	C	C	C	C	C	L	R	C	C	C	0	0	0	0	0	0	0	0	0	0	-1	0	0
106	Pull up H2O to wash C18	3	0	400	0	800	-200	0	0	0	0	0	0	0	0	0	1	0	C	C	C	C	C	C	C	C	C	C	R	C	C	C	C	C	C	C	C	C	L	R	C	C	C	0	0	0	0	0	0	0	0	0	0	-1	0	0
107	Pull up H2O to wash C18	3	0	200	0	800	-200	0	0	0	0	0	0	0	0	0	1	0	C	C	C	C	C	C	C	C	C	C	R	C	C	C	C	C	C	C	C	C	L	R	C	C	C	0	0	0	0	0	0	0	0	0	0	-1	0	0
108	Pull up H2O to wash C18	3	0	0	0	800	-200	0	0	0	0	0	0	0	0	0	1	0	C	C	C	C	C	C	C	C	C	C	R	C	C	C	C	C	C	C	C	C	L	R	C	C	C	0	0	0	0	0	0	0	0	0	0	-1	0	0
109	Dry C18 Plus Long	10	0	0	0	800	-200	0	0	0	0	1	0	0	0	0	1	0	C	C	C	C	C	C	C	C	C	C	C	C	C	C	C	C	C	C	C	C	L	R	C	C	C	0	0	0	0	0	0	0	0	0	0	-1	0	0

Steps 110 through 147

Product elution into final dose vial

110	Dry Line	15	0	0	0	800	-1000	0	0	0	0	1	0	0	0	0	1	0	C	C	C	C	C	C	C	C	C	C	C	C	C	C	C	C	C	C	C	C	C	C	C	C	C	0	0	0	0	0	0	0	0	0	2	-1	0	0
111	Pull EtOH to elute C18	3	0	30	0	800	-200	0	0	0	0	0	0	0	0	0	1	0	C	C	C	C	C	C	C	C	C	C	R	C	C	C	C	C	C	C	C	C	C	C	C	C	C	0	0	0	0	0	0	0	0	70	1	-1	0	0
112	Pull EtOH to elute C18	7	0	100	0	800	-200	0	0	0	0	0	0	0	0	0	1	0	C	C	C	C	C	C	C	C	C	C	R	C	L	C	C	C	C	C	C	C	L	R	C	C	L	0	0	0	0	0	0	0	0	0	0	-1	0	0
113	Pull EtOH to elute C18	7	0	600	0	800	-200	0	0	0	0	1	0	0	0	0	1	0	C	C	C	C	C	C	C	C	C	C	L	C	C	C	C	C	C	C	C	C	L	R	C	C	L	0	0	0	0	0	0	0	0	0	0	-1	0	0
114	Elute EtOH in PV	3	0	550	0	800	-200	0	0	0	0	0	0	0	0	0	1	0	C	C	C	C	C	C	C	C	C	C	R	C	C	C	C	C	C	C	C	C	L	R	C	C	L	0	0	0	0	0	0	0	0	0	0	-1	0	0
115	Elute EtOH in PV	3	0	500	0	800	-200	0	0	0	0	0	0	0	0	0	1	0	C	C	C	C	C	C	C	C	C	C	R	C	C	C	C	C	C	C	C	C	L	R	C	C	L	0	0	0	0	0	0	0	0	0	0	-1	0	0
116	Elute EtOH in PV	3	0	450	0	800	-200	0	0	0	0	0	0	0	0	0	1	0	C	C	C	C	C	C	C	C	C	C	R	C	C	C	C	C	C	C	C	C	L	R	C	C	L	0	0	0	0	0	0	0	0	0	0	-1	0	0
117	Elute EtOH in PV	3	0	400	0	800	-200	0	0	0	0	0	0	0	0	0	1	0	C	C	C	C	C	C	C	C	C	C	R	C	C	C	C	C	C	C	C	C	L	R	C	C	L	0	0	0	0	0	0	0	0	0	0	-1	0	0
118	Elute EtOH in PV	3	0	350	0	800	-200	0	0	0	0	0	0	0	0	0	1	0	C	C	C	C	C	C	C	C	C	C	R	C	C	C	C	C	C	C	C	C	L	R	C	C	L	0	0	0	0	0	0	0	0	0	0	-1	0	0
119	Elute EtOH in PV	3	0	300	0	800	-200	0	0	0	0	0	0	0	0	0	1	0	C	C	C	C	C	C	C	C	C	C	R	C	C	C	C	C	C	C	C	C	L	R	C	C	L	0	0	0	0	0	0	0	0	0	0	-1	0	0
120	Elute EtOH in PV	3	0	250	0	800	-200	0	0	0	0	0	0	0	0	0	1	0	C	C	C	C	C	C	C	C	C	C	R	C	C	C	C	C	C	C	C	C	L	R	C	C	L	0	0	0	0	0	0	0	0	0	0	-1	0	0
121	Elute EtOH in PV	3	0	200	0	800	-200	0	0	0	0	0	0	0	0	0	1	0	C	C	C	C	C	C	C	C	C	C	R	C	C	C	C	C	C	C	C	C	L	R	C	C	L	0	0	0	0	0	0	0	0	0	0	-1	0	0
122	Elute EtOH in PV	3	0	150	0	800	-200	0	0	0	0	0	0	0	0	0	1	0	C	C	C	C	C	C	C	C	C	C	R	C	C	C	C	C	C	C	C	C	L	R	C	C	L	0	0	0	0	0	0	0	0	0	0	-1	0	0
123	Elute EtOH in PV	3	0	100	0	800	-200	0	0	0	0	0	0	0	0	0	1	0	C	C	C	C	C	C	C	C	C	C	R	C	C	C	C	C	C	C	C	C	L	R	C	C	L	0	0	0	0	0	0	0	0	0	0	-1	0	0
124	Elute EtOH in PV	3	0	50	0	800	-200	0	0	0	0	0	0	0	0	0	1	0	C	C	C	C	C	C	C	C	C	C	R	C	C	C	C	C	C	C	C	C	L	R	C	C	L	0	0	0	0	0	0	0	0	0	0	-1	0	0
125	Elute EtOH in PV	3	0	0	0	800	-200	0	0	0	0	0	0	0	0	0	1	0	C	C	C	C	C	C	C	C	C	C	R	C	C	C	C	C	C	C	C	C	L	R	C	C	L	0	0	0	0	0	0	0	0	0	0	-1	0	0
126	Elute EtOH in PV	10	0	0	0	800	-200	0	0	0	0	1	0	0	0	0	1	0	C	C	C	C	C	C	C	C	C	C	C	C	C	C	C	C	C	C	C	C	L	R	C	C	L	0	0	0	0	0	0	0	0	0	0	-1	0	0
127	Pull NS elution of C18 to PV	3	0	30	0	800	-200	0	0	0	0	0	0	0	0	0	1	0	C	C	C	C	C	C	C	C	C	C	R	C	C	C	C	C	C	C	C	C	C	C	C	C	C	0	0	0	0	0	0	0	0	0	0	-1	0	0
128	Pull NS elution of C18 to PV	7	0	600	0	800	-200	0	0	0	0	0	0	0	0	0	1	0	C	C	C	C	C	C	C	C	C	C	R	C	C	C	C	L	C	C	C	C	L	R	C	C	L	0	0	0	0	0	0	0	0	0	0	-1	0	0
129	Elute NS in PV	3	0	550	0	800	-200	0	0	0	0	0	0	0	0	0	1	0	C	C	C	C	C	C	C	C	C	C	R	C	C	C	C	C	C	C	C	C	L	R	C	C	L	0	0	0	0	0	0	0	0	0	0	-1	0	0
130	Elute NS in PV	3	0	500	0	800	-200	0	0	0	0	0	0	0	0	0	1	0	C	C	C	C	C	C	C	C	C	C	R	C	C	C	C	C	C	C	C	C	L	R	C	C	L	0	0	0	0	0	0	0	0	0	0	-1	0	0
131	Elute NS in PV	3	0	450	0	800	-200	0	0	0	0	0	0	0	0	0	1	0	C	C	C	C	C	C	C	C	C	C	R	C	C	C	C	C	C	C	C	C	L	R	C	C	L	0	0	0	0	0	0	0	0	0	0	-1	0	0
132	Elute NS in PV	3	0	400	0	800	-200	0	0	0	0	0	0	0	0	0	1	0	C	C	C	C	C	C	C	C	C	C	R	C	C	C	C	C	C	C	C	C	L	R	C	C	L	0	0	0	0	0	0	0	0	0	0	-1	0	0
133	Elute NS in PV	3	0	350	0	800	-200	0	0	0	0	0	0	0	0	0	1	0	C	C	C	C	C	C	C	C	C	C	R	C	C	C	C	C	C	C	C	C	L	R	C	C	L	0	0	0	0	0	0	0	0	0	0	-1	0	0
134	Elute NS in PV	3	0	300	0	800	-200	0	0	0	0	0	0	0	0	0	1	0	C	C	C	C	C	C	C	C	C	C	R	C	C	C	C	C	C	C	C	C	L	R	C	C	L	0	0	0	0	0	0	0	0	0	0	-1	0	0
135	Elute NS in PV	3	0	250	0	800	-200	0	0	0	0	0	0	0	0	0	1	0	C	C	C	C	C	C	C	C	C	C	R	C	C	C	C	C	C	C	C	C	L	R	C	C	L	0	0	0	0	0	0	0	0	0	0	-1	0	0
136	Elute NS in PV	3	0	200	0	800	-200	0	0	0	0	0	0	0	0	0	1	0	C	C	C	C	C	C	C	C	C	C	R	C	C	C	C	C	C	C	C	C	L	R	C	C	L	0	0	0	0	0	0	0	0	0	0	-1	0	0
137	Elute NS in PV	3	0	150	0	800	-200	0	0	0	0	0	0	0	0	0	1	0	C	C	C	C	C	C	C	C	C	C	R	C	C	C	C	C	C	C	C	C	L	R	C	C	L	0	0	0	0	0	0	0	0	0	0	-1	0	0
138	Elute NS in PV	3	0	100	0	800	-200	0	0	0	0	0	0	0	0	0	1	0	C	C	C	C	C	C	C	C	C	C	R	C	C	C	C	C	C	C	C	C	L	R	C	C	L	0	0	0	0	0	0	0	0	0	0	-1	0	0
139	Elute NS in PV	3	0	50	0	800	-200	0	0	0	0	0	0	0	0	0	1	0	C	C	C	C	C	C	C	C	C	C	R	C	C	C	C	C	C	C	C	C	L	R	C	C	L	0	0	0	0	0	0	0	0	0	0	-1	0	0
140	Elute NS in PV	3	0	0	0	800	-200	0	0	0	0	0	0	0	0	0	1	0	C	C	C	C	C	C	C	C	C	C	R	C	C	C	C	C	C	C	C	C	L	R	C	C	L	0	0	0	0	0	0	0	0	0	0	-1	0	0
141	Elute NS in PV	15	0	0	0	800	-200	0	0	0	0	1	0	0	0	0	1	0	C	C	C	C	C	C	C	C	C	C	C	C	C	C	C	C	C	C	C	C	L	R	C	C	L	0	0	0	0	0	0	0	0	0	0	-1	0	0
142	Start of cassette rinse	1	0	0	0	800	-200	0	0	0	0	0	0	0	0	0	0	1	C	C	C	C	C	C	C	C	C	C	C	C	C	C	C	C	C	C	C	C	C	C	C	C	C	0	0	0	0	0	0	0	0	80	0	-1	0	0
143	Emptying water bottle	15	0	0	0	800	-500	0	0	0	0	1	0	0	0	0	1	0	C	C	C	C	C	C	C	C	C	C	C	C	C	C	C	C	C	C	C	C	C	C	C	C	C	0	0	0	0	0	0	0	0	0	2	-1	0	0
144	Flushing manifold	10	0	0	0	800	-200	0	0	0	0	1	0	0	0	0	1	0	C	C	C	C	C	C	C	C	C	C	C	C	C	C	C	C	C	C	C	C	C	C	C	C	C	0	0	0	0	0	0	0	0	0	1	-1	0	0
145	Event	1	0	0	0	0	0	0	0	0	0	0	0	0	0	0	0	0	C	C	C	C	C	C	C	C	C	C	C	C	C	C	C	C	C	C	C	C	C	C	C	C	C	0	0	0	0	0	0	0	0	0	1	-1	0	10
146	END	3	0	0	0	800	0	0	0	0	0	0	0	0	0	0	0	0	C	C	C	C	C	C	C	C	C	C	C	C	C	C	C	C	C	C	C	C	C	C	C	C	C	0	0	0	0	0	0	0	0	0	3	-1	0	0
147	END	1	0	0	0	800	0	0	0	0	0	0	0	0	0	0	0	0	C	C	C	C	C	C	C	C	C	C	C	C	C	C	C	C	C	C	C	C	C	C	C	C	C	0	0	0	0	0	0	0	0	90	0	-1	0	0

Step 111 – A 100 mm syringe displacement units (SDU) on the 5 mL syringe is equivalent to 0.52 mL of EtOH (200 proof)

Step 127 – A 600 mm syringe displacement units (SDU) on the 5 mL syringe (with a 4.4 mL NS vial) is equivalent to 4.2 mL of NS

Figure S2: **Representitive images for needle placement of the LAH vial when punctured and attached onto the FASTLab Cassette.**

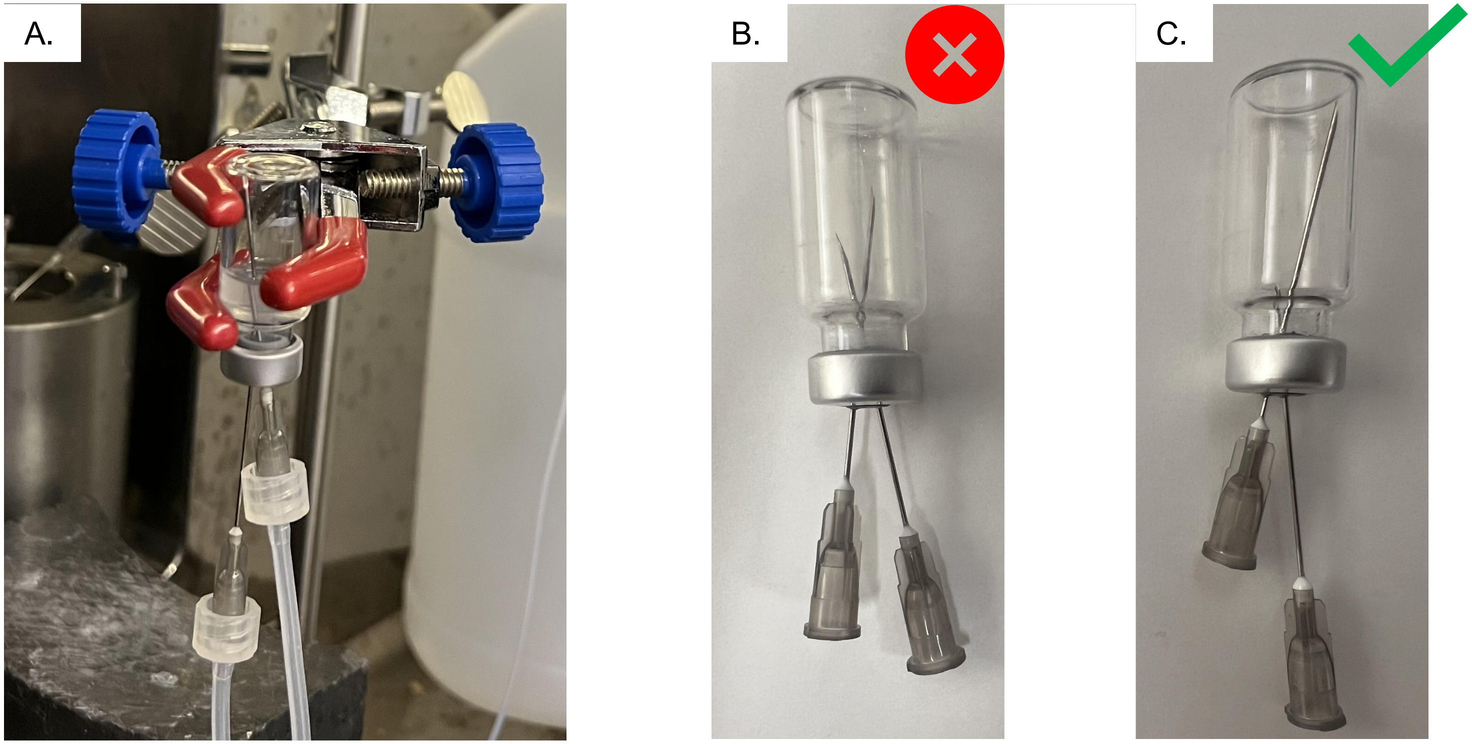

Figure S2a – Shows the needle placement and the arrangement of the inverted LAH vial. Note that the needle that is above the solvent line (when inverted) should be attached to V1, corresponding to the nitrogen overpressure, and the needle that is submerged in the solvent, but slightly above the septa (when inverted) should be attached to V4, corresponding to where LAH will be drained.
Figure S2b – shows the incorrect placement of needles (the solvent is removed for increased clarity)
Figure S2c – shows the correct placement of needles where one of the needles is right around the shoulder of the inverted vial (the solvent is removed for increased clarity)

1. When puncturing the vial, have the vial upright and insert the first needle and tubing connected to V1 all the way down to the bottom of the vial into the LAH solution
2. Puncture the vial with the second needle and tubing connected to V4 close to where the septa and neck is.
3. Slowly invert the vial such that the needle connected to V4 is submerged and the needle and V1 is above the solvent line near the bottom of the vial (but when the vial is inverted the needle is facing the “top”).
4. Clamp the inverted vial, or tape the inverted vial.

Figure S3: Representative analytical HPLC chromatographs using two analytical methods for [^{11}C]butanol. Sample HPLC traces for [^{11}C]butanol using the new Luna C18(2) column method.

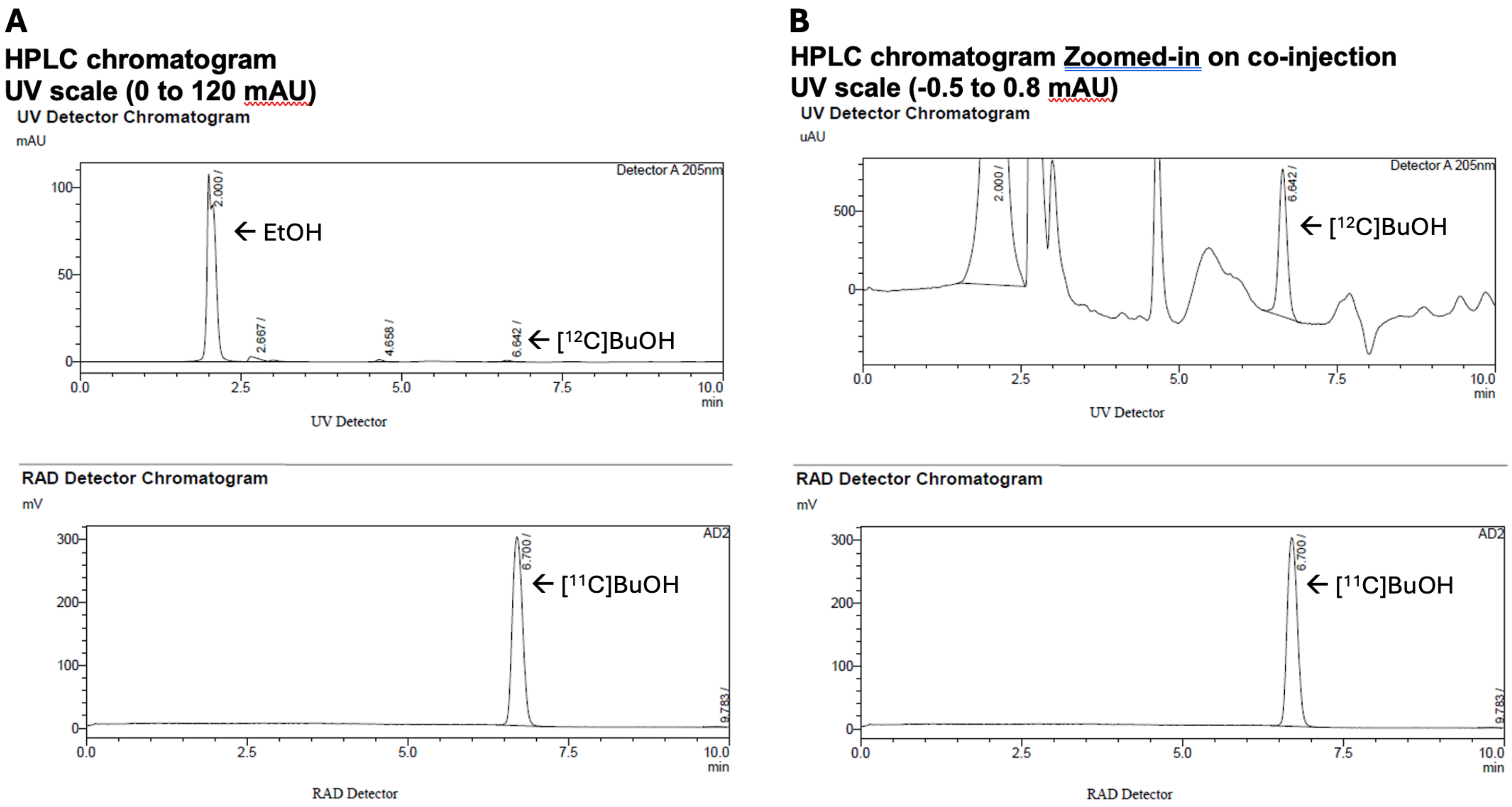

Figure S3a – New HPLC method [column: Phenomenex Luna C18(2), 5 µm, 100 Å, 250 x 4.6 mm; mobile phase: 20% acetonitrile; flow rate: 1.2 mL/min; oven: 40 °C; UV detection at 205 nm*; t_R butanol ~6.5 min]

Figure S3b – Zoom of Fig 3a showing co-injected reference standard

* We recorded UV at 205 nm as this was the internally approved method from our previous studies conducted on the TracerLab. 205 nm was able to detect butanol with the best limit of detection. Other UV settings were investigated, but resulted in a higher limit of detection which was not ideal.

Figure S4: Representative analytical HPLC chromatographs using the previously reported Rezex RCM-monosaccharide Ca^{+2} column method

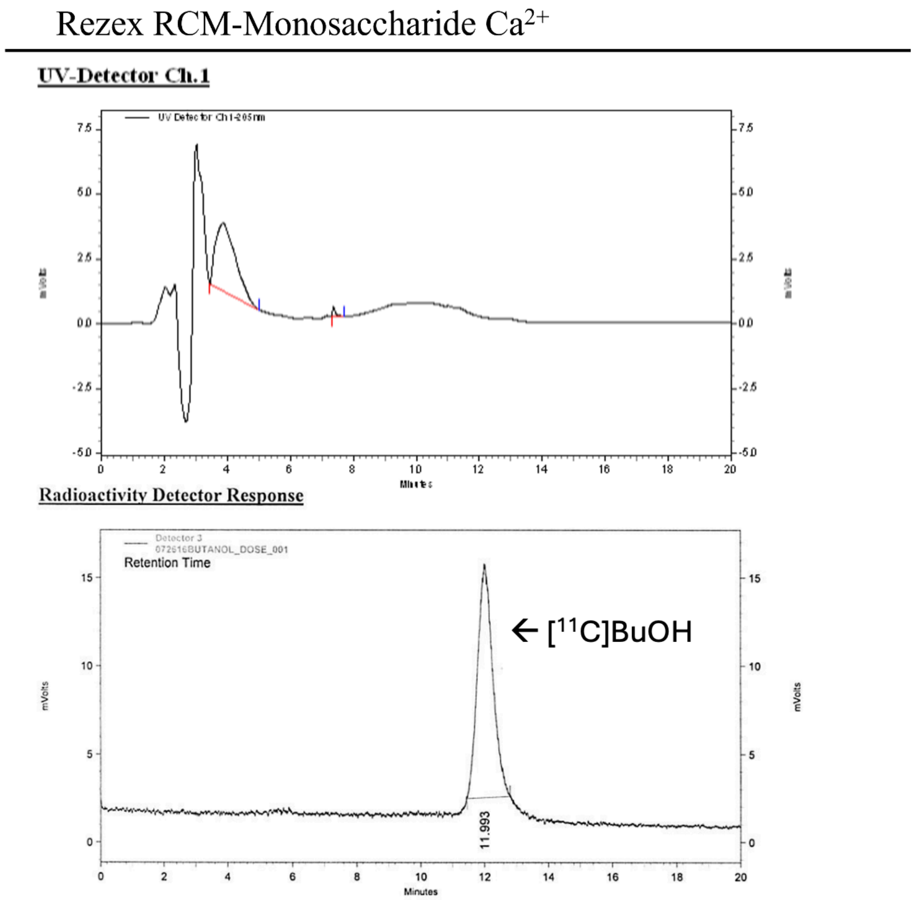

Previously reported HPLC method [column: Phenomenex Rezex RCM-Monosaccharide Ca^{+2}, 300 x 7.8 mm; mobile phase: 100% water; flow rate: 1.4 mL/min; oven: 60 °C; UV detection at 205 nm†; t_R butanol ~12 min]

† We recorded UV at 205 nm as this was the internally approved method from our previous studies conducted on the TracerLab. 205 nm was able to detect butanol with the best limit of detection. Other UV settings were investigated, but resulted in a higher limit of detection which was not ideal.
